# Land-Use Intensity Rather Than Plant Functional Identity Shapes Bacterial and Fungal Rhizosphere Communities

**DOI:** 10.3389/fmicb.2018.02711

**Published:** 2018-11-20

**Authors:** Ricardo Schöps, Kezia Goldmann, Katharina Herz, Guillaume Lentendu, Ingo Schöning, Helge Bruelheide, Tesfaye Wubet, François Buscot

**Affiliations:** ^1^Department of Soil Ecology, UFZ – Helmholtz-Centre for Environmental Research, Halle, Germany; ^2^Department of Biology II, Leipzig University, Leipzig, Germany; ^3^Institute of Biology/Geobotany and Botanical Garden, Martin Luther University Halle-Wittenberg, Halle, Germany; ^4^Department of Ecology, University of Kaiserslautern, Kaiserslautern, Germany; ^5^Max Planck Institute for Biogeochemistry, Jena, Germany; ^6^German Centre for Integrative Biodiversity Research (iDiv) Halle-Jena-Leipzig, Leipzig, Germany

**Keywords:** microbial composition, alpha-diversity, land-use intensity, temperate grassland, next-generation sequencing, bacterial 16S, fungal ITS2

## Abstract

The rhizosphere encompasses the soil surrounding the surface of plants’ fine roots. Accordingly, the microbiome present is influenced by both soil type and plant species. Furthermore, soil microbial communities respond to land-use intensity due to the effects on soil conditions and plant performance. However, there is limited knowledge about the impact of grassland management practices under field conditions on the composition of both bacteria and fungi in the rhizosphere of different plant functional groups. In spring 2014 we planted four phytometer species, two forbs (*Plantago lanceolata*, *Achillea millefolium*) and two grasses (*Dactylis glomerata*, *Arrhenatherum elatius*) into 13 permanent experimental grassland plots, differing in management. After 6 months, rhizosphere and bulk soil associated with the phytometer plants were sampled, microbial genomic DNA was extracted and bacterial 16S and fungal ITS rDNA were sequenced using Illumina MiSeq. Our study revealed that the rhizosphere microbial community was more diverse than the bulk soil community. There were no differences in microbial community composition between the two plant functional groups, but a clear impact of root traits and edaphic conditions. Land-use intensity strongly affected plant productivity, neighboring plant richness and edaphic conditions, especially soil C/N ratio, which in turn had a strong influence on root traits and thereby explained to large extent microbial community composition. Rhizosphere microbes were mainly affected by abiotic factors, in particular by land-use intensity, while plant functional type had only subordinate effects. Our study provides novel insights into the assembly of rhizosphere bacterial and fungal communities in response to land-use intensity and plant functional groups in managed grassland ecosystems.

## Introduction

Soil microbial communities play a major role in biogeochemical cycles by influencing carbon and nutrient cycling (van der Heijden et al., 2008). Thus, they affect ecosystem functioning directly (Bardgett and Van Der Putten, 2014). Plants, as primary producers, rely on nutrient exchange with soil microbes; hence their rhizospheres constitute hotspots of microbial activity (Bakker et al., 2013). A reduced microbial diversity in the rhizosphere compared to bulk soil, considered to be equivalent to a microbial seed bank, was reported recently (Philippot et al., 2013). In contrast, some studies have also demonstrated increased microbial diversity in the rhizosphere compared to the bulk soil (Dawson et al., 2017; Novello et al., 2017). These contradictions highlight the complexity of habitat-microbe relationships and the need for further investigations.

Of the microbial groups in the rhizosphere, bacteria and fungi are important members since their functions range from symbionts and pathogens to decomposers. Previous studies revealed that especially fast-growing bacteria like the phyla *Proteobacteria*, particularly *Alpha-*, *Betaproteobacteria*, and *Bacteroidetes*, are major groups in the rhizosphere community (Berg and Smalla, 2009; Turner et al., 2013; Oberholster et al., 2018). For fungi, Ascomycota, especially the order Hypocreales, form the majority of the rhizosphere inhabitants (Mouhamadou et al., 2013; Philippot et al., 2013). However, in comparison to bacteria, general fungi are currently underrepresented in rhizosphere studies.

Microbes of the rhizosphere community favor nutrient-rich conditions in which plants provide available carbon through secretion of photosynthates. They correspond to roughly 10% of the photosynthetically fixed carbon and 15% of total plant nitrogen (Venturi and Keel, 2016). Plants can specifically select their rhizosphere microbiome via these root exudates – the so-called ‘rhizosphere effect’ (Berendsen et al., 2012): It has been shown that amount and type of root exudates, which vary between plant species, influence rhizosphere microbial communities (Costa et al., 2006; Ladygina and Hedlund, 2010; Burns et al., 2015). Conversely, other studies were unable to find any plant identity effect (Nunan et al., 2005; Singh et al., 2007). Different plant functional groups like grasses and forbs have distinct characteristics and fill distinct niches (Roscher et al., 2004; Herz et al., 2017a). Relative to forbs, grasses have a higher belowground biomass leading to dense root systems (Siebenkäs et al., 2015; Ravenek et al., 2016). This results in higher litter decomposition rates and enhanced soil nutrient cycling in grass communities (Wu et al., 2011). Consequently, different plant functional groups are likely to promote distinct microbial groups.

Recent experiments on the effect of plant functional groups on microbial community composition only considered bulk soil (König et al., 2010; Dassen et al., 2017). This excluded the identification of plant–microbe interactions from these studies (Barea et al., 2005). In general, plant traits, especially those belowground, may explain an important proportion of microbial community dynamics in the rhizosphere (Eisenhauer and Powell, 2017). Currently, knowledge of these interactions originates from laboratory studies (Thion et al., 2016). Such experiments have either focused on microbial biomass as a proxy for microbial communities (Steinauer et al., 2017) or on specific groups like nitrogen-related microorganisms (Legay et al., 2014). Comparable field studies evaluating the impact of belowground plant traits on microbial communities, are still scarce (Bardgett et al., 2014).

Besides biotic factors, different soil types as a measure of soil quality are assumed to harbor specific microbial communities (Berg and Smalla, 2009). These differences of microbial assemblage might be caused either through the direct effect of soil properties (Wang et al., 2009) or indirectly by belowground plant traits, e.g., root exudation (Berg and Smalla, 2009). Moreover, soil properties such as pH, soil carbon, and nutrient contents can even be altered by anthropogenic influence, e.g., through fertilization (Lauber et al., 2008; Herzog et al., 2015). There is evidence that land-use intensity can shift bacterial community composition (Kaiser et al., 2016; Estendorfer et al., 2017). Fertilization in combination with increased disturbance caused by grazing and/or mowing is responsible for microbial changes (Gossner et al., 2016). Even bacteria at high taxonomic levels exhibit management preferences: *Acidobacteria* is consistently associated with little-managed soils, whereas *Actinobacteria*, *Beta*-, and *Gammaproteobacteria* are often found under fertilized conditions (Herzog et al., 2015; Francioli et al., 2016; Ho et al., 2017). For fungi, fertilized soils have positive effects on Mucoromycota (formerly Zygomycota), in particular on the genus *Mortierella* (Francioli et al., 2016). In contrast, the genera *Camarophyllopsis* and *Cuphophyllus* are associated with nutrient-poor grasslands (Lodge et al., 2014). However, the majority of studies on land-use or management effects have compared very different ecosystems, such as forests, grasslands and arable fields (Lauber et al., 2008; Thomson et al., 2015; Tian et al., 2017). With the exception of a study on arbuscular mycorrhizal fungi (AMF) in roots (Vályi et al., 2015), yet there is no study on the microbial response of rhizospheric communities in grasslands across different management regimes and land-use intensities.

The aim of the present study was to assess to which extent bacterial and fungal communities in the rhizosphere and bulk soil are influenced by plant functional group, plant traits and land-use intensity. Therefore, rhizosphere and bulk soil samples were taken from forb and grass phytometers planted in 13 experimental plots with different land-use intensities within the “German Biodiversity Exploratories” (Fischer et al., 2010). We applied paired-end amplicon sequencing of the bacterial 16S rRNA and fungal ITS2 regions using Illumina MiSeq. Bioinformatic and statistical tools were used to assess the diversity and composition of bacterial and fungal communities. We postulated that (i) due to the rhizosphere effect, we expect distinct microbiomes in soil surrounding the roots compared to the bulk soil. We further hypothesized that (ii) the rhizosphere microbiome varies according to functional group and traits of plants. Finally, we tested whether (iii) land-use intensity shapes the bulk and rhizosphere microbial communities. We expected a weak or even suppressed rhizosphere effect under high land-use intensity.

## Materials and Methods

### Study Site

The study was carried out in Central Germany in the Hainich National Park and its surroundings (Hainich-Dün, ca. 1, 300 km^2^; 51°16′N10°47′E) within the German Biodiversity Exploratories project (Fischer et al., 2010). In total, 13 out of the 50 experimental grassland plots (dimensions: 50 m × 50 m) were chosen; these represented three different land-use types: meadow, mown pasture, and pasture. Each land-use type corresponds to a specific regime of mowing, grazing and fertilization, which have been quantified and combined to create land-use intensity indices (LUI, Blüthgen et al., 2012). This classification allows simultaneous comparisons between all experimental plots. This study used mean LUI values for 2014, ranging from 0.58 to 2.66. Low values indicate extensive and high values intensive management regimes. The main soil types in the experimental plots are Cambisol, Vertisol, and Stagnosol [according to the Food and Agriculture Organization (FAO) soil classification system; Supplementary Table S1]. In addition, plant aboveground biomass, equivalent to plant productivity, was derived from the 2014 vegetation survey (Klaus et al., 2016). Briefly, aboveground biomass was harvested in four randomly placed quadrates of 0.25 m^2^. Shrubs and dead plant litter were excluded from biomass sampling. The material from the four quadrates was pooled, dried for 48 h at 80°C and weighted to the nearest gram. The selected soil variables were: pH, carbon to nitrogen ratio (soil C/N ratio), total phosphorus (TP, Schöning et al., 2013; Solly et al., 2014; Richter et al., 2018), and Olsen plant available (NaHCO_3_-extractable) phosphorous (PAP, Olsen, 1954; Alt et al., 2011). The values of the environmental variables for each experimental plot are listed in Supplementary Table S1.

### Phytometer Plant Preparation and Sample Collection

Our setup considered a 2 × 4 factorial experimental design with two soil compartments (bulk versus rhizosphere soil) and four phytometer plant species (2 forbs (*Plantago lanceolata* L. and *Achillea millefolium* L.) + 2 grasses (*Arrhenatherum elatius* (L.) P. Beauv. ex J. Presl & C. Presl and *Dactylis glomerata* L. s. str.)) and was conducted in 13 experimental plots. Phytometer plants were prepared in the following way: seeds of two perennial forbs (*P. lanceolata* and *A. millefolium*) and two perennial grasses (*A. elatius* and *D. glomerata*) were collected from all of the 50 Hainich experimental plots in 2011 and 2013. These seeds were sown in 5.5 cm × 5.5 cm pots containing a 1:1 silt and sand mixture in December 2013 in the greenhouse of the Botanical Garden in Halle (Saale), Germany. Conditions in the greenhouse were 20°C during daytime and 10°C during the night with a 12 h/12 h day/night rhythm. The obtained plant seedlings were randomly transferred outside as phytometers into the 13 experimental plots with different LUI values in May and early June 2014 (see Herz et al., 2017a). The silt-sand mixture from the pre-cultivated plants was removed by washing the roots with tap water. Next, these phytometer plants were planted directly into the soil of each experimental plot. This phytometer plant approach (Dietrich et al., 2013) was used because it allowed us a complete harvest of roots in a large set of experimental plots. As all phytometer plants were raised under the same conditions and were of the same age, this is a suitable approach to minimize unwanted, random variation and to gain comparable results. In addition, the phytometer plant approach allowed us to compare all target species across all target experimental grassland plots (Herz et al., 2017a,b). The first monitoring of the establishment of the phytometer plants and replacement planting of individuals that died due to transplantation shock took place from mid-May to June 2014. At the same time and in addition to the data from the vegetation survey (plant productivity per experimental plot) we estimated the cover and richness of the neighboring vegetation in a circle of 15 cm radius around each phytometer (Herz et al., 2017a,b). Each individual of the four plant species planted into each of the 13 experimental plots was harvested in September 2014. This autumn sampling allowed sufficient regrowth of the phytometers after mowing or grazing during the vegetation season. The phytometer plants were excavated along with monoliths of soil measuring about 20 cm × 20 cm × 20 cm surrounding their root systems. In each monolith, the soil only loosely attached to the roots, which could be separated by hand shaking, was considered to represent the bulk soil. In contrast, the soil still adhering to the roots after shaking was gently brushed away and collected as rhizosphere soil. The bulk and rhizosphere soil fractions were immediately flash frozen in liquid nitrogen in the field, and stored on dry ice until their transfer into −80°C freezers in the laboratory. In addition, the phytometers were individually separated into roots, shoots and leaves, and further processed to measure several above and belowground traits (for detailed information see Herz et al., 2017a,b). In total, 104 samples were collected for the analysis of microbial communities {13 experimental plots × 4 plant species [2 forbs (*P. lanceolata* and *A. millefolium*) + 2 grasses (*A. elatius* and *D. glomerata*)] × 2 soil compartments (rhizosphere and bulk soil)}. A short description of all plant traits and plot variables examined is given in Supplementary Table S2.

### DNA Extraction, Library Preparation and Multiplexing

Soil microbial genomic DNA was extracted from each of the bulk and rhizosphere soil samples using a PowerSoil DNA Isolation Kit (MO BIO Laboratories Inc., Carlsbad, CA, United States) according to the slightly modified manufacturer’s instructions. We used 0.4 g instead of 0.25 g of soil for the extractions. DNA yields from each sample were checked with a NanoDrop ND-8000 spectrophotometer (Thermo Fisher Scientific, Dreieich, Germany), and the extracts were stored at −20°C. DNA extracts were adjusted to 10–15 ng/μl. The bacterial 16S rRNA gene V4 region was amplified using the universal primer pair 515f and 806r (Caporaso et al., 2011) with Illumina adapter sequences. All PCRs were conducted using the proofreading Kapa Hifi polymerase (Kapa Biosystems, Boston, MA, United States). The following thermal profile was used: initial denaturation at 95°C for 3 min, 25 cycles of denaturation at 98°C for 20 s, annealing at 55°C for 15 s, elongation at 72°C for 15 s and a final extension at 72°C for 5 min. To generate the fungal amplicon library, nested PCRs were performed, starting with amplification of the fungal ITS1 and ITS2 rDNA region using the primer combination ITS1F (Gardes and Bruns, 1993) and ITS4 (White et al., 1990). PCR thermo-cycle conditions were as follows: initial denaturation at 95°C for 5 min, 10 cycles of denaturation at 98°C for 20 s, annealing at 50–60°C for 15 s (−1°C per cycle), followed by elongation at 72°C for 15 s and 2 cycles of denaturation at 98°C for 20 s, annealing at 50°C for 15 s, followed by elongation at 72°C for 15 s. The final extension was carried out at 72°C for 5 min. The ITS2 region was subsequently amplified using 1:10 diluted products of the first PCR and the primer pair fITS7 (Ihrmark et al., 2012) and ITS4 (White et al., 1990) containing the Illumina adapter sequences. PCR was performed under the following conditions: initial denaturation at 95°C for 5 min, 25 cycles of denaturation at 98°C for 20 s, annealing at 56°C for 15 s, followed by elongation at 72°C for 15 s and a final extension at 72°C for 5 min.

The amplicon libraries created were checked by gel electrophoresis and purified with an Agencourt AMPure XP kit (Beckman Coulter, Krefeld, Germany). Illumina Nextera XT Indices were added to both ends of the bacterial and fungal fragments in the next PCR. The thermal profile was as follows: initial denaturation at 95°C for 3 min, 8 cycles of denaturation at 98°C for 30 s, annealing at 55°C for 30 s, followed by elongation at 72°C for 30 s and a final extension at 72°C for 5 min. Finally, products were purified with AMPure beads. Bacterial and fungal libraries were quantified by PicoGreen assays (Molecular Probes, Eugene, OR, United States) and then pooled in one tube to give equimolar representation of each. Fragment sizes and quality of DNA sequencing libraries were determined by Agilent 2100 Bioanalyzer (Agilent Technologies, Palo Alto, CA, United States). Paired-end sequencing of 2 × 300 bp of this pool was performed using a MiSeq Reagent kit v3 on an Illumina MiSeq platform (Illumina Inc., San Diego, CA, United States) at the Department of Soil Ecology, Helmholtz Centre of Environmental Research [UFZ, Halle (Saale), Germany]. The raw 16S and ITS rDNA sequences were deposited in the National Center for Biotechnology Information (NCBI) Sequence Read Archive (SRA) under study accession number SRP133002.

### Bioinformatics Workflow

Raw forward and reverse reads were demultiplexed with default parameters (mismatch = 1) by the Illumina reporter software v2.5.1.3 according to the index combinations, and provided as fastq files with the Illumina adaptors, indices and sequencing primers removed. Further bioinformatic processing was carried out on a high performance computing (HPC) cluster using custom bash scripts. Pair-end reads were merged using PandaSeq v2.8.1 with a minimum overlap of 20 and a threshold of 0.6 (Masella et al., 2012). Reads shorter than 200 nt, with any ambiguous nucleotide or with homopolymers of 10 nt or longer were removed using MOTHUR v1.39.5 (Schloss et al., 2009). Pre-clustering was performed in order to reduce the computational workload and to filter out reads resulting from sequencing errors by allowing only a maximum of 1% dissimilarity using cd-hit-454 v4.6.1 (Niu et al., 2010). Potential chimeric sequences were discarded after a chimera check using UCHIME in *de novo* mode as implemented in MOTHUR (Schloss et al., 2009; Edgar et al., 2011). The remaining reads from each sample were pooled, de-replicated into unique sequences and sorted by decreasing abundance using OBITools v1.2.11 (Boyer et al., 2016). The retained high-quality reads were clustered into operational taxonomic units (OTUs) at 97% similarity using cd-hit-est v4.6.1 (Fu et al., 2012). A second chimera check was conducted on OTU representative sequences using UCHIME in *de novo* mode and putative chimeric OTUs were removed. OTU-representative sequences were classified for bacterial 16S against the SILVA database v128 (2016-11-28, Quast et al., 2013) and for fungal ITS against the UNITE database v7 (2016-01-31, Kõljalg et al., 2013) using the Bayesian classifier as implemented in MOTHUR (Schloss et al., 2009). After removal of singletons, doubletons and tripletons, a total of 7,602,424 bacterial and 2,434,456 fungal quality filtered sequences were obtained from 104 samples (52 bulk and 52 rhizosphere soil samples). Afterwards, plant derived 16S sequences that were assigned to chloroplasts or mitochondria were removed from the bacterial OTU table. Fungal references sequences were checked additionally with ITSx v1.0.11 (Bengtsson-Palme et al., 2013) to be ITS2 sequences from fungi. The detected non-fungal sequences were removed from downstream analysis. Zygomycota and Glomeromycota classification were changed to Mucoromycota according to Spatafora et al. (2016). Sample reads were normalized for bacteria to 31,000 and for fungi to 10,000 by using the function “rarefy_even_depth” from the phyloseq package 1.19.1 (McMurdie and Holmes, 2013) in R v3.4.2 (R Core Team, 2017). Functional annotation of all bacterial OTUs from the normalized data was parsed against the FAPROTAX (v1.1; Louca et al., 2016b) database to assign putative life strategies to taxonomically defined OTUs. The database has been modified from its original version by choosing relevant functions for our study and by integrating plant beneficial functional groups based on literature survey: plant growth promoting rhizobacteria (PGPR), fungal/bacterial antagonists, nematocidal activity, siderophore production, phytohormone production, phosphate solubilizing bacteria and associative nitrogen fixation. In addition, potential fungal functional groups were assigned to the fungal OTUs from the normalized data where possible using the online annotation tool: FUNGuild (Nguyen et al., 2016).

### Statistical Analysis

**FIGURE 1:**
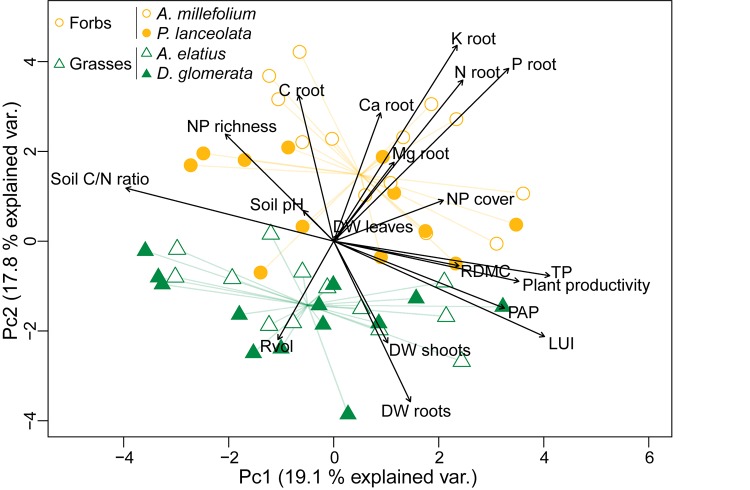
Principal component analysis (PCA) plot showing the multivariate variation among 104 samples in terms of plant traits and environmental variables. Vectors indicate the direction and strength of each plant trait and environmental variable to the overall distribution. Colored symbols correspond to the two plant functional groups (grasses versus forbs) defined in this study.

The statistical analyses were conducted in R v3.4.2 (R Core Team, 2017) and in PAST v2.17c (Hammer et al., 2001). Initially, we conducted separate analyses to test for the effects of plant identity or functional group. As there were no differences between plant species (i.e., plant identity had no significant effect) and since the plant traits clearly separate the phytometer plant species based on their functional groups (Figure 1), we only report the analyses including plant functional group. Principal components analysis (PCA) was conducted with ranked variables to test relationships between the biotic and abiotic variables using ‘prcomp’ function from the stats package.

Bacterial and fungal OTU richness and abundance-based coverage estimator (ACE), as alpha-diversity indices, were calculated using vegan (Oksanen et al., 2017) and fossil (Vavrek, 2011), respectively. To identify whether alpha-diversity (richness and ACE) were affected by the fixed factors soil compartment, plant species/plant functional group or LUI, we applied linear mixed effect models (LMEM, packages lmer, Bates et al., 2015 and lmerTest, Kuznetsova et al., 2017). Experimental plot and soil type were considered as random factors; marginal and conditional *R*^2^-values were calculated to evaluate goodness-of-fit of the model using the ‘r.squaredGLMM’ function (Nakagawa and Schielzeth, 2013). The best model was identified as that with the lowest AICc (Akaike’s information criterion for small sample sizes). Multiple mean comparisons using Tukey’s test were performed to determine how bacterial and fungal alpha-diversity differed between soil compartments by using the ‘glht’ function of R package multcomp (version 1.4-7, (Hothorn et al., 2008). Relationships between bacterial and fungal OTU richness, respectively, and biotic and abiotic variables were calculated using non-parametric Spearman’s rank correlation.

Further correlations of bacterial and fungal communities with soil compartments, plant functional groups, biotic and abiotic factors were visualized by means of non-metric multidimensional scaling (NMDS) on the basis of Bray–Curtis distance and relative abundance data. The significant biotic and abiotic variables (*p* < 0.05) were fitted as vectors into the NMDS ordination plots using the ‘envfit’ function in the vegan package and Goodness-of-fit statistics (*R*^2^) were calculated based on 999 permutations (Oksanen et al., 2017). Variation in bacterial and fungal community composition explained by soil compartment (or separately for each compartment), plant functional group, LUI, and the interaction between them were tested for significance using the Bray–Curtis dissimilarities and permutational analysis of variance (PERMANOVA, ‘adonis’ function in the R package ‘vegan,’ Oksanen et al., 2017). To estimate the source of variation for bacterial and fungal communities and to compare the effects of the biotic and abiotic factors we used variation partitioning (varpart function in vegan). Thus, models were constructed containing four groups of predictors: soil compartment (rhizosphere vs. bulk soil), plant functional group (forbs vs. grasses), plant traits and plot variables (Supplementary Table S2).

Similarity percentages (SIMPER) analysis based on Bray-Curtis dissimilarity and the relative abundance of all bacterial or fungal genera was used to calculate the pairwise and overall dissimilarity between the rhizosphere and bulk soil using PAST (Hammer et al., 2001). We extracted the top 30 bacterial and fungal genera that contributed the most to the observed overall dissimilarity (Supplementary Table S3) for further analyses.

Since microbial abundance data is often dominated by zeros (zero-inflated), we applied generalized joint attribute modeling (gjam) v2.1.8 (Clark et al., 2017) to test for differential abundance of the most abundant bacterial and fungal phyla (i.e., each phylum with at least 1% relative abundance) or the top 30 bacterial and fungal genera in the rhizosphere versus bulk soil or with different LUI. The posterior simulation was produced by Gibbs sampling and this analysis was based on composition count data (‘CC’). The different microbial functions and functional groups retained from FAPROTAX and FUNGuild were also tested for differential abundance in the rhizosphere versus bulk soil by applying gjam.

Models of multivariate analysis of variance were constructed using partial distance-based redundancy analysis (db-RDA) based on the Bray–Curtis distance with the ‘capscale’ function in vegan to determine the biotic and abiotic variables that were most influential on the bacterial and fungal community compositions. We accounted for soil type effects before testing the constraints by including them as source of conditional variation. Abiotic and biotic factors were standardized to a constant mean and standard deviation using ‘scale’ function in R. We first verified whether there was co-linearity between factors using the ‘varclust’ function in the Hmisc package (Harrell and Dupont, 2017) and then performed a stepwise model selection using permutation tests with ‘ordistep’ function (vegan). These procedures were undertaken using both the full dataset (rhizosphere and bulk soil) and separately for each soil compartment.

With the DESeq2 package in R, a differential analysis of the microbial OTUs in the rhizosphere or bulk soil of forbs versus grasses was conducted using moderated shrinkage estimation for dispersions and fold changes as an input for a pairwise Wald test (Love et al., 2014). This test evaluates the number of bacterial or fungal OTUs significantly enriched in plant functional groups in the different soil compartments (*p* < 0.05). For these analyses we used the un-rarefied OTU counts as normalization is implemented in the DESeq2 package (Oberholster et al., 2018).

To define the ecological niche of the top 30 microbial genera, abundance-weighted means (AWMs) of LUI for each genus were calculated. This analysis was done at genus level because more accurate information can be retrieved at this lower taxonomic levels, which then leads to better predictions of putative ecological roles in the respective systems (Hartmann et al., 2014). The AWMs and the abundance-weighted standard deviation (AWSD) of the LUI were assessed by means of the ‘wtd.mean’ and square root of the ‘wtd.var’ function in the Hmisc package (Harrell and Dupont, 2017). Further, we calculated the coefficients of variation (CV) by dividing the standard deviation with the mean AWMs of all and the top 30 microbial genera to account for the variability in their ecological niche preference.

## Results

### Diversity and Characterization of Bacterial and Fungal Datasets

The rarefaction curves obtained from the bacterial and fungal data sets approached saturation. This indicates that our sequencing depths were sufficient (Supplementary Figure S1). The normalization procedure resulted in 18,446 bacterial and 4,841 fungal OTUs. We were able to assign 93, 75, 66, 47, and 28% of the bacterial and 89, 71, 66, 58, and 47% of the fungal OTUs to the phylum, class, order, family and genus levels, respectively. The most abundant bacterial phyla (i.e., each phylum with at least 1% relative abundance) in both the rhizosphere and bulk soils were *Proteobacteria* (classes *Alpha*-, *Beta*-, *Gamma*, and *Deltaproteobacteria*), followed by *Actinobacteria, Acidobacteria*, *Planctomycetes*, *Verrucomicrobia*, *Bacteroidetes*, *Chloroflexi*, *Gemmatimonadetes*, *Firmicutes*, *Nitrospirae*, *Latescibacteria* and unclassified bacteria (Supplementary Figure S2). Among the fungi, Ascomycota, followed by Basidiomycota, Mucoromycota (Mortierellomycotina, 7% and Glomeromycotina, 3%), Chytridiomycota and unclassified fungi were the most abundant phyla in both compartments (Supplementary Figure S2). Based on FAPROTAX, a total of 3,820 bacterial OTUs (∼22%) could be assigned to at least one functional group. In addition, we assigned 2,472 fungal OTUs (∼51% from all 4,841 fungal OTUs) to functional groups. The fungal functional unassigned OTUs with the highest abundance had a proportion below 2%. The unassigned and assigned OTUs were similarly distributed across the data set.

### Relationships Between the Biotic and Abiotic Variables

Principal component analysis (PCA) showed plant traits, abiotic factors and their relationships between the plant functional groups (Figure 1). The first axis (Pc1) was mainly characterized by abiotic factors and the second axis (Pc2) by plant traits. Plant traits especially root macronutrients were dependent on each other (*p* < 0.05, Supplementary Table S4) but showed no significant correlation with LUI (*p* > 0.05). The grasses were characterized by root volume, root and shoot dry weight, while the forbs were characterized by high macronutrient contents (C, P, K, Ca root content). Root dry matter content (RDMC) was similar between the plant functional groups.

### Bulk Versus Rhizosphere Soil

**FIGURE 2:**
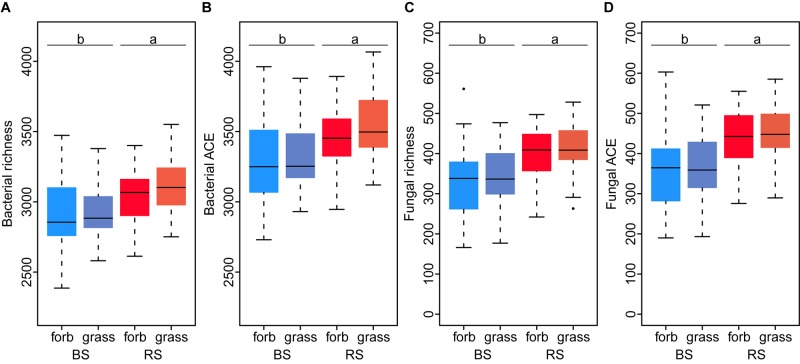
Bacterial **(A,B)** and fungal **(C,D)** OTU richness and abundance-based coverage estimator (ACE) as a function of plant functional group (grass versus forb species) and soil compartment [rhizosphere soil (RS) versus bulk soil (BS)].

**FIGURE 3:**
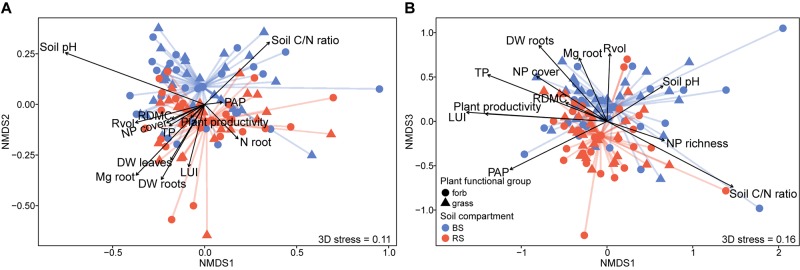
Non-metric multidimensional scaling (NMDS) ordination of bacterial **(A)** and fungal **(B)** community composition in rhizosphere (red) and bulk (blue) soil both under forbs (•) and grasses (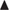) based on Bray–Curtis dissimilarity and *k* = 3 dimensions. Significant vectors (*p* < 0.05) correlated with community composition are shown. BS, bulk soil; RS, rhizosphere soil; LUI, land-use intensity index; DW leaf/root, dry weight root/leaf; RDMC, root dry matter content; Rvol, root volume; Mg root, root magnesium content; N root, root nitrogen content; Soil C/N ratio, soil carbon to nitrogen ratio; PAP, plant available phosphorus; TP, soil total phosphorus; Plant productivity, plant biomass per plot; NP richness/cover, richness/cover of the neighboring plants.

**FIGURE 4:**
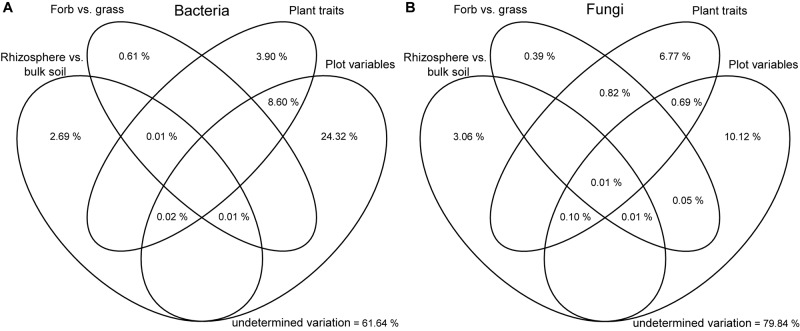
Variation partitioning analysis, illustrating the effects of soil compartment, plant functional group, plant traits and plot variables on the community structure of bacteria **(A)** and fungi **(B)**. Each ellipse represents the portion of variation accounted by each factor. Shared variance is represented by the intersecting portions of the ellipses. Values ≤ 0 are not shown.

For the analysis of the alpha-diversity with fixed factors, we used a model without interactions, which was identified as the best model (lowest AICc) in the model selection. Analysis of bacterial and fungal alpha-diversity revealed significant differences between the rhizosphere and bulk soil. A higher observed and estimated OTU richness was found in the rhizosphere compartment (Figure 2 and Supplementary Tables S5, S6). Fitting the bacterial and fungal beta-diversity patterns using NMDS revealed two slightly distinct clusters between rhizosphere and bulk soil communities (Figure 3 and Supplementary Figure S3). The PERMANOVA analysis confirmed the effect of the soil compartment on bacterial (*p* < 0.001) and fungal (*p* < 0.001) community composition at the OTU level (Table 1). Overall, the soil compartment explained comparable amounts of variance for both microbial groups (2.69% for bacteria and 3.06% for fungi; Figure 4).

**Table 1:** Effect of soil compartment, plant functional group and land-use intensity (LUI) on bacterial and fungal OTU community compositions assessed with permutational multivariate analysis of variance (PERMANOVA).

Bacteria	Total			Bulk soil			Rhizosphere soil		
	df	*F*-value	*R*^2^	df	*F*-value	*R*^2^	df	*F*-value	*R*^2^
Soil compartment (SC)	1	3.18^∗∗∗^	0.03						
Functional group (FG)	1	1.00	0.01	1	0.90	0.01	1	0.59	0.01
Land-use intensity (LUI)	1	5.24^∗∗∗^	0.05	1	3.13^∗∗∗^	0.06	1	2.82^∗∗∗^	0.05
SC × LUI	1	0.49	0.01						
SC × FG	1	0.72	0.01						
FG × LUI	1	0.72	0.01	1	0.63	0.01	1	0.44	0.01
SC × FG × LUI	1	0.36	0.00						
Residuals	94		0.89	47		0.92	47		0.92
**Fungi**									
Soil compartment (SC)	1	3.90^∗∗∗^	0.03						
Functional group (FG)	1	0.60	0.01	0.56	0.02	1	0.50	0.01
Land-use intensity (LUI)	1	5.13^∗∗∗^	0.05	3.05^∗∗∗^	0.06	1	2.72^∗∗∗^	0.06
SC × LUI	1	0.47	0.01						
SC × FG	1	0.64	0.01						
PS × LUI	1	0.46	0.01	0.38	0.01	1	0.41	0.01
SC × FG × LUI	1	0.33	0.00						
Residuals	94		0.89		0.92	47		0.92

*Soil compartment (rhizosphere soil vs. bulk soil); plant functional group (forbs vs. grasses); df, degrees of freedom; ^∗∗∗^p < 0.001*.

**FIGURE 5:**
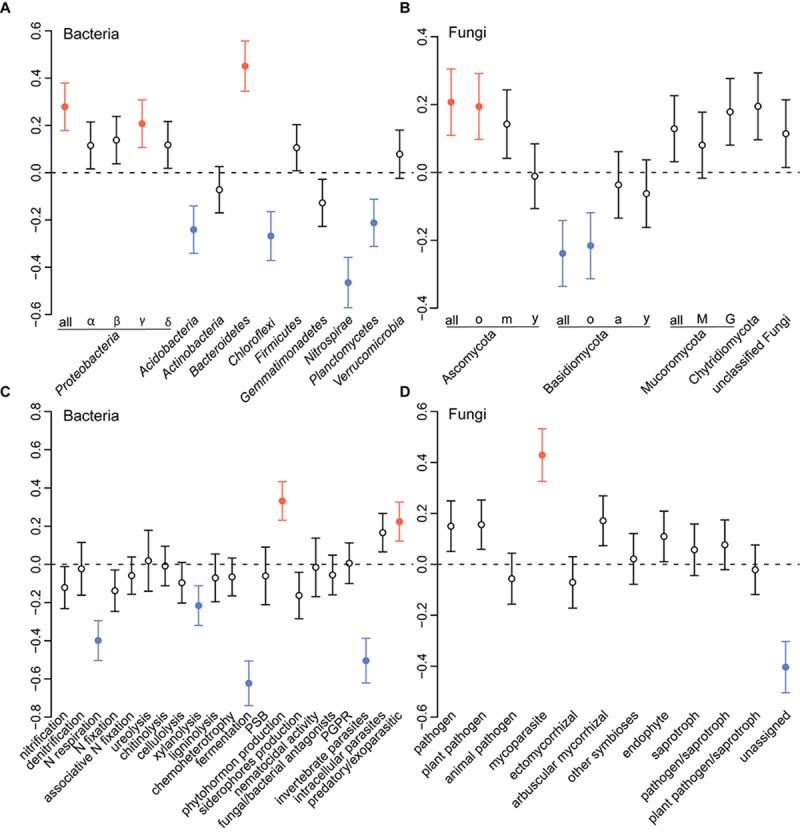
Posterior mean (with standard deviation) estimates of standardized coefficients (dimensionless) showing the variation in bacterial **(A,C)** and fungal **(B,D)** phyla and functional group OTU abundance between the rhizosphere and bulk soil. Red or blue colors indicate strong positive or negative responses of microbial relative abundance to the rhizosphere soil tested with generalized attribute modeling (gjam). **(A,B)** All, entire OTU abundance of a phyla; o, other Ascomycota and Basidiomycota species; m, ascomycetes mold fungi; y, ascomycetes and basidiomycetes yeast species; a, agaricoid basidiomycetes; M, Mortierellomycotina; G, Glomeromycotina subphyla of Mucoromycota. **(C)** PSB, phosphate solubilizing bacteria; PGPR, plant growth promoting rhizobacteria.

For bacterial phyla, the rhizosphere soil displayed a higher relative OTU abundance for *Proteobacteria* in total and for the class *Gammaproteobacteria* and *Bacteroidetes* (Figure 5A). In the rhizosphere, there was reduced OTU abundance for *Acidobacteria*, *Chloroflexi*, *Nitrospirae*, and *Planctomycetes*. The major differences for fungal phyla between the two soil compartments were reduced relative abundance of OTUs from Basidiomycota and other basidiomycetes species (not sorted in the groups of agaricoid basidiomycetes or yeasts) in the rhizosphere. In contrast, OTU abundances of Ascomycota and other ascomycetes species (not sorted into the groups of ascomycetes mold fungi or yeasts) were higher in the rhizosphere (Figure 5B).

The functional assignment revealed changes in the abundance of OTUs related to bacterial and fungal ecological groups between the soil compartments (Figures 5C,D). Thereby, several fungal functional groups tend to have a higher abundance within the rhizosphere soil whereas most bacterial functional groups were equally distributed within the soil compartments or showed tendencies to be enhanced in bulk soil. However, two bacterial functional groups, i.e., the one of predatory/exoparasitic bacteria and the one related to phytohormon production, showed an increased abundance in the rhizosphere (red color). In contrast, functional groups related to the nitrogen respiration, xylanolysis, fermentation and invertebrate parasites were more often found in the bulk soil (blue color). For fungi, only mycoparasites were clearly more abundant in the rhizosphere whereas fungi without functional assignment were often found in the bulk soil.

### Impact of Plant Functional Group and Root Traits

Overall, there was no influence of the plant functional groups on microbial alpha-diversity (Supplementary Table S6). In addition, no interactions between soil compartment and plant functional group occurred in the models with the smallest AICc.

Moreover, plant functional group had no direct influence on the microbial community composition, a fact that was also true when the rhizosphere and bulk soil communities were considered separately (Table 1). In total, only a small part of the variation could be exclusively explained by plant-related factors, accounting for 4 and 7% of bacterial and fungal variance, respectively (Figure 4). In addition, for bacteria, the shared fraction of plant traits and plot accounted for 9%.

**FIGURE 6:**
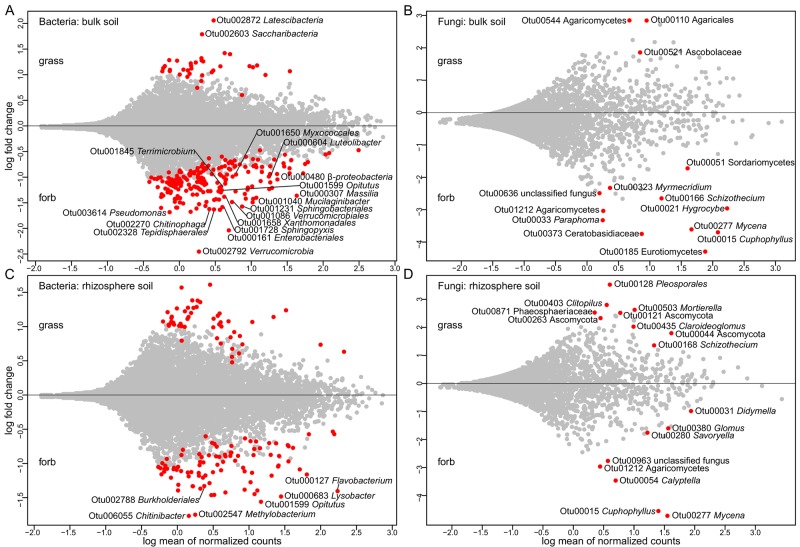
Pairwise comparisons of the plant functional groups grasses and forbs in different soil compartments (rhizosphere versus bulk soil) for bacterial **(A–C)** and fungal **(B–D)** OTUs. In each experimental plot, the shapes depict individual OTUs whose position on the *x*-axis reflects the abundance (normalized counts) and the position on the *y*-axis the fold change in the indicated comparison. OTUs with a significant fold change are highlighted in red (Wald test, *p* < 0.05). Taxonomic affiliation labels have been added for relevant OTUs (*p* < 0.001 for bacteria and *p* < 0.05 for fungi; full list of significant OTUs are shown in Supplementary Table S7).

**FIGURE 7:**
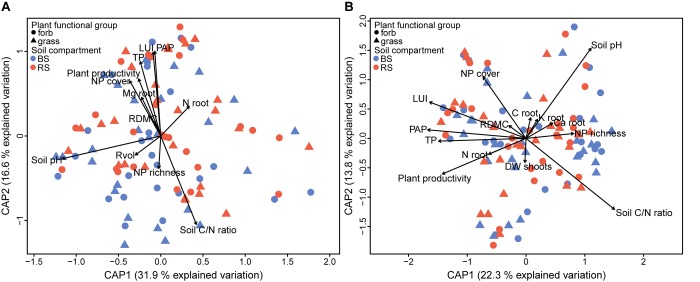
Partial distance-based redundancy analysis (db-RDA) for the bacterial **(A)** and fungal **(B)** communities associated with the plant traits and environmental factors based on Bray–Curtis dissimilarity. BS, bulk soil; RS, rhizosphere soil; LUI, land-use intensity index; DW leaf/root, dry weight root/leaf; RDMC, root dry matter content; Rvol, root volume; Mg root, root magnesium content; N root, root nitrogen content; Soil C/N ratio, soil carbon to nitrogen ratio; PAP, plant available phosphorus; TP, soil total phosphorus; Plant productivity, plant biomass per experimental plot; NP richness/cover, richness/cover of the neighboring plants.

For more detailed insights, we performed pairwise comparisons to identify those bacterial and fungal OTUs that differed significantly between the plant functional groups in the rhizosphere and bulk soil by using DESeq2 (Figure 6 and Supplementary Table S7). In agreement with the above analyses, the vast majority of microbial OTUs were shared between forbs and grasses (Wald test, *p* > 0.05). However, a higher number of individual bacterial OTUs were found in the bulk soil around forbs (204 OTUs) compared to grasses (35 OTUs), while in the rhizosphere soil 94 OTUs and 51 OTUs differentiate forbs and grasses, respectively (Wald test, *p* < 0.05). Regarding the fungi, only three and 11 OTUs were enriched in the bulk soil under grasses and forbs, respectively. In rhizosphere soil, we observed comparable numbers of significantly abundant OTUs under grasses (9 OTUs) and forbs (8 OTUs). In total, no marked differential effects between the plant functional groups on OTU richness of bacteria and fungi or on their community composition and repartition in microbial functional groups could be found within the two soil compartments (Supplementary Figures S4, S5 and Supplementary Table S7). However, a profound effect of various root traits on the bacterial and fungal communities was revealed by partial db-RDA: RDMC and root N content had an impact on microbial community composition (Figure 7 and Table 2). In addition, bacterial community composition responded also to root volume and root Mg content, while fungal community composition was correlated with shoot dry weight, root C, P, Ca, and K content. Interestingly, the bacterial rhizosphere community was not affected by root traits. In contrast, in bulk soil, the bacterial community reacted to RDMC and root N content (Table 2 and Supplementary Figure S6). The fungal bulk soil community responded to root N and K content and the rhizosphere community to RDMC.

**Table 2:** The most influential factors affecting bacterial and fungal OTU community composition as determined by partial distance-based redundancy analysis (db-RDA).

	Bacteria						Fungi					
	Total		BS		RS		Total		BS		RS	
	*F*	*p*	*F*	*p*	*F*	*p*	*F*	*p*	*F*	*p*	*F*	*p*
DW leaves												
DW shoot							1.58	^∗^				
DW root												
RDMC	1.73	^∗^	1.73	^∗^			1.95	^∗∗^			2.13	^∗∗^
Rvol	1.65	^∗^										
C root							1.68	^∗^				
N root	1.78	^∗^	1.73	^∗^			1.57	^∗^	1.43	^∗^		
P root												
Mg root	1.66	^∗^										
Ca root							1.46	^∗^				
K root							1.84	^∗∗^	1.38	^∗^		
LUI	4.91	^∗∗∗^	2.82	^∗∗^	3.22	^∗∗∗^	4.12	^∗∗∗^	2.20	^∗∗∗^	2.41	^∗∗∗^
Soil pH	11.90	^∗∗∗^	6.81	^∗∗∗^	6.80	^∗∗∗^	5.38	^∗∗∗^	2.80	^∗∗∗^	3.40	^∗∗∗^
Soil C/N ratio	8.57	^∗∗∗^	5.20	^∗∗∗^	5.46	^∗∗∗^	7.08	^∗∗∗^	4.03	^∗∗∗^	3.95	^∗∗∗^
PAP	4.44	^∗∗∗^	2.45	^∗∗^	3.15	^∗∗∗^	3.13	^∗∗∗^	1.94	^∗∗∗^	1.68	^∗^
TP	4.91	^∗∗∗^	3.56	^∗∗^	3.28	^∗∗∗^	4.72	^∗∗∗^	2.29	^∗∗∗^	2.66	^∗∗∗^
Plant productivity	5.74	^∗∗∗^	3.61	^∗∗∗^	2.63	^∗∗∗^	3.97	^∗∗∗^	2.76	^∗∗∗^	2.38	^∗∗^
NP richness	3.65	^∗∗∗^	2.32	^∗∗^	1.79	^∗^	2.37	^∗∗∗^	1.62	^∗∗^		
NP cover	2.85	^∗∗∗^	1.91	^∗^	2.33	^∗∗^	1.86	^∗∗^				

*Blank fields show removed factors by model selection using ordistep function in R; DW leaf/shoot/root, dry weight leaf/shoot/root; RDMC, root dry matter content; Rvol, root volume; C root, root carbon content; N root, root nitrogen content; P root, root phosphorus content; Mg root, root magnesium content; Ca root, root calcium content; K root, root potassium content; LUI, land-use intensity index; Soil C/N ratio, soil carbon to nitrogen ratio; PAP, plant available phosphorus; TP, soil total phosphorus; Plant productivity, plant biomass per experimental plot; NP richness/cover, richness/cover of the neighboring plants; ^∗^p < 0.05, ^∗∗^p < 0.01, ^∗∗∗^p < 0.001.*

### Effect of Land-Use Intensity and Other Biotic and Abiotic Factors

Microbial richness was not influenced by land-use intensity (Supplementary Table S6). However, LUI significantly affected bacterial and fungal community composition (Figures 3, 7 and Table 1). PERMANOVAs carried out separately for rhizosphere and bulk soil indicated that LUI explained about 6% of the variance in both the bacterial and fungal communities (Table 1). Variance partitioning (Figure 4) showed that the largest part of the community variation was exclusively explained by plot-related variables such as LUI, soil physicochemical properties and soil type for both bacteria (24%) and fungi (10%). Overall, a larger part of the total community variance could be explained for the bacterial (38%) than for the fungal communities (20%).

**FIGURE 8:**
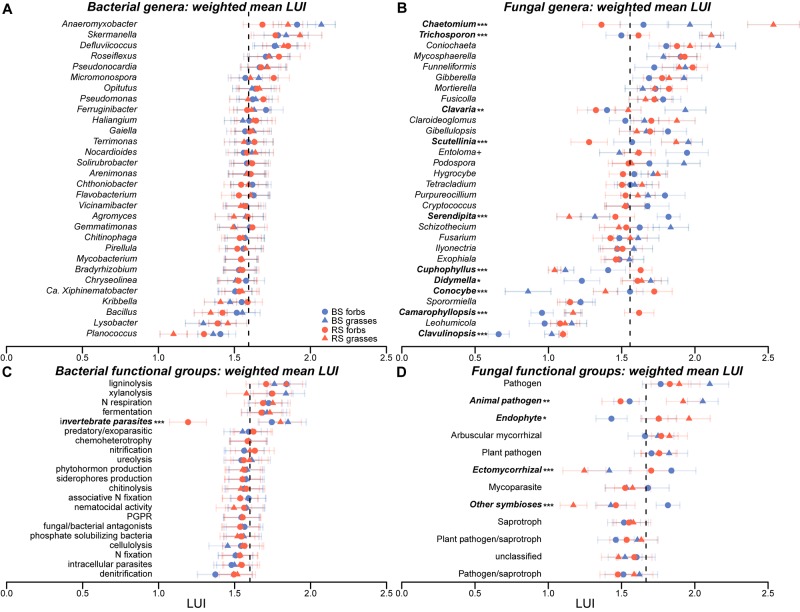
Abundance-weighted means (AWM) of land-use intensity for the top 30 bacterial **(A)** and fungal **(B)** genera that contributed the most to the dissimilarities as well as bacterial **(C)** and fungal **(D)** functional groups in rhizosphere and bulk soil of forbs and grasses along with weighted standard errors. Dotted line illustrates the AWM of land-use intensity across all genera and microbial functional groups found. Differences between the rhizosphere and bulk soil of forbs and grasses are indicated by: ^+^*p* < 0.10, ^∗^*p* < 0.05, ^∗∗^*p* < 0.01, ^∗∗∗^*p* < 0.001.

The AWM LUI were calculated at genus level for bacteria and fungi because this lower taxonomic level contains more information. The average AWM LUI across all genera was 1.59 (standard deviation = 0.29) for bacteria and 1.56 (standard deviation = 0.42) for fungi but not statistically different (*t* = 1.432, *p* = 0.152; Figure 8 and Supplementary Figure S7). The top 30 bacterial genera occurred in a narrower range of land-use intensity (1.10–2.07) than did fungal genera (0.66–2.54). This higher variability of fungi is also reflected when calculating the CV for all bacterial (CV = 18%) and fungal genera (CV = 27%) as well as for the top 30 bacterial (CV = 8%) and fungal genera (CV = 19%). In addition, while tolerance to LUI did not vary significantly between soil compartments and plant functional groups for bacteria, we found more profound differences for fungi. In particular, the fungal genera of the top 30 that were found at the upper and lower limit had a highly variable specificity to LUI in the soil compartments and plant functional groups. In contrast, bacteria showed a uniform pattern with hardly any differences between soil compartments and plant functional groups. For either the rhizosphere or bulk soil, the generalized attribute models identified bacterial and fungal genera that were positively or negatively related to LUI (Figure 8 and Supplementary Figure S8). The pattern that fungal genera have a higher variability to LUI between the soil compartments and plant functional groups than bacterial genera is also shown at the microbial functional level (CV of 5% for bacterial and 8% for fungal functions, Supplementary Figure S9).

As the PCA implies (Figure 1), Spearman rank correlation analysis of LUI against soil C/N ratio, and neighboring plant richness revealed strong negative correlations (ρ = −0.56, *p* < 0.001 and ρ = −0.63, *p* < 0.001, respectively; Supplementary Table S4), while plant available phosphorus (ρ = 0.36, *p* = 0.010), soil total phosphorus (ρ = 0.40, *p* = 0.003) and plant productivity (ρ = 0.52, *p* < 0.001) were positively affected by LUI. The partial db-RDA model identified abiotic and biotic factors which explained the changes in bacterial and fungal community composition (Figure 7 and Table 2). In particular, soil pH (*F* = 11.90, *p* < 0.001 for bacteria and *F* = 5.38, *p* < 0.001 for fungi) and C/N ratio (*F* = 8.57, *p* < 0.001 for bacteria and *F* = 7.08, *p* < 0.001 for fungi) were the soil physicochemical properties having the strongest influence on both bacterial and fungal community composition. This strong effect of soil pH and C/N ratio was also evident individually for bacterial and fungal rhizosphere and bulk soil communities (Table 2 and Supplementary Figure S6). Interestingly, the cover of the directly neighboring plants from our phytometer species seemed to influence only the bacteria in the rhizosphere (*F* = 2.33, *p* = 0.007) and bulk soil (*F* = 1.92, *p* = 0.021), while richness of the directly neighboring plants was correlated with the bacterial rhizosphere (*F* = 1.79, *p* = 0.019) and bulk soil (*F* = 2.32, *p* = 0.006) as well as with the fungal bulk soil communities (*F* = 1.62, *p* = 0.007).

## Discussion

### Differences in Microbial Diversity and Composition for the Two Soil Compartments Examined

Our results demonstrated a different rhizosphere assemblage and diversity compared to the surrounding bulk soil. We found a clear increase in microbial richness from the bulk soil to the rhizosphere, which is in contrast to observations of a lower alpha-diversity in the root-associated soil than the bulk soil (Xu et al., 2012; Pii et al., 2016; Wang et al., 2017). Nevertheless, our results are consistent with some existing literature (Edwards et al., 2015; Dawson et al., 2017; Novello et al., 2017). Differences between the soil compartments have not been discussed in these previous studies. A possible reason for a higher rhizosphere than bulk soil diversity could be that the microbial communities in the rhizosphere are more differentiated and contain a high number of specialists (Mendes et al., 2014). Such specific microbes are also present in the initial species pool of the bulk soil but, due to their rarity they are under the detection limit of the molecular methods we used (Hugerth and Andersson, 2017). In addition, we sampled the two compartments toward the end of the vegetation period. At this time, plants provide a pulse of readily available carbon substrates into soils (Bardgett et al., 2005). Differences in the composition of these compounds may promote the diversity of the microbial community and lead to strong niche differentiation (Hinsinger et al., 2005).

It is noteworthy, that the overall explanatory power of the two microbial communities between the two soil compartments was low, amounting to 2.80% for bacteria and 3.14% for fungi, respectively. This weak effect may correspond to the 6 months of phytometer growth, which may not have been sufficient for clear ecological differentiation between the rhizosphere and bulk soil compartments. We can anticipate that the rhizosphere effect would be more pronounced in subsequent years (Smalla et al., 2001). The difference between the two habitats is also inherently limited by the fact that rhizosphere communities are basically recruited from the surrounding bulk soil (Berendsen et al., 2012). Furthermore, plants in grasslands are tightly linked by mycelial networks (Wick et al., 2007; Ellouze et al., 2014), which can mitigate community differences between rhizosphere and bulk soil. Despite all these factors that tend to homogenize the communities of the two soil compartments, we found typical rhizosphere bacterial phyla such as *Proteobacteria* and *Bacteroidetes* enriched in the proximity of roots. These are considered to be fast-growing copiotrophic organisms (Fierer et al., 2007), relying on labile carbon compounds provided by root exudation. In addition and consistent with the known beneficial effect of several groups of the *Proteobacteria* for plants (Hayat et al., 2010), we also found bacteria that synthesize and export phytohormones playing positive roles in plant growth and development predominantly in the rhizosphere. However, other plant beneficial groups with an expected occurrence in the rhizosphere were found to be in equal proportions in both soil compartments. In contrast, we observed a higher abundance of *Acidobacteria* and *Nitrospirae* in bulk soil compared to the rhizosphere. These phyla are considered to be slow growing and are assumed to have an oligotrophic lifestyle (Daims et al., 2015; Fierer, 2017). *Acidobacteria* are generally considered to have a preference for oligotrophic bulk soil (Bulgarelli et al., 2013; Peiffer et al., 2013; Schreiter et al., 2014), while the poorly studied *Nitrospirae* have previously been found to be more abundant in the rhizosphere (Lopes et al., 2016; Oberholster et al., 2018).

Regarding the fungal phyla, our findings revealed a preference of Ascomycota for rhizosphere soil and Basidiomycota for bulk soil, which is in line with previous reports (Xu et al., 2012; Mouhamadou et al., 2013). Although it is generally difficult to assign a common life strategy to an entire phylum, there is evidence that basidiomycetes degrade more complex C substrates while ascomycetes exhibit copiotrophic characteristics and respond quickly to root exudates (Philippot et al., 2013; Ho et al., 2017). Assigning ecological functions to various taxa tends to a slightly higher abundance of neutral and detrimental fungal functional groups in the rhizosphere compared with the bulk soil. But in particular, the putative plant mutualistic mycoparasites were strongly abundant in the rhizosphere. These fungi may suppress and/or inhibit plant pathogens and thus, mycoparasites are considered as biological control agents (Kim and Vujanovic, 2016). We found a general rhizosphere effect with differences in diversity and community composition compared to the bulk soil, despite our phytometer plants only growing for 6 months in the field. Further, the enriched microbial functional groups are consistent with the specific ecological life conditions in the rhizosphere.

### Effects of Plant Functional Group and Traits on Rhizosphere Microbiome

Numerous studies have clearly shown that plant species identity shapes the structure of rhizosphere-associated microbial populations (Costa et al., 2006; Berg and Smalla, 2009; Berendsen et al., 2012). Thus, we expected individual patterns for the investigated plant functional groups. However, we only found small differences for individual OTUs and no clear distinction with respect to microbial community composition and diversity between the plant functional groups. This finding is partly consistent with a recent study, including grasses, tall and small herbs and legumes, which found no effect on bacterial and fungal communities in non-rhizosphere soil (Dassen et al., 2017). Those authors only found distinct communities of particular groups of soil microorganisms, such as AMF between different plant functional groups which is in agreement with López-García et al. (2017). Our finding of a general rhizosphere effect but without differences between the investigated plant functional groups and plant species identity therein (Supplementary Figures S4, S5), suggests that forbs and grasses may exude equivalent compounds (Millard and Singh, 2010; Chaparro et al., 2013). Hence, rhizosphere communities may be primarily shaped by a general copiotrophic lifestyle rather than by a dependence on specific exudate compounds. However, this absence of differential response may reflect the timeframe after planting out the phytometers, as specific groups of pathogen or beneficial functional microbes may take longer to invade roots (Berendsen et al., 2012) or establish in the rhizosphere (Fierer, 2017). The influence of plants is comparatively small relative to that of the soil physiochemical environment and the LUI (Millard and Singh, 2010). Obviously, at least after 6 months of out planting the phytometers into the field, a potential plant functional group effect was missing and might be hidden within the strength of environmental effects explaining 24.32 and 10.12% of the observed variation for bacterial and fungal communities, respectively. Though we assume that with ongoing time the plant effect might increase, the effect of LUI can continue to dominate the plant functional group effect as fertilization and the frequent disturbance by mowing and grazing continue homogenizing the microbial community composition.

Plant trait-based approaches have recently been used to describe shifts in the abundances and the functional characteristics of microbial communities (Eisenhauer and Powell, 2017). With our study, we are able to link plant traits with the general bacterial and fungal community composition. In particular, some root morphological traits together with root chemical properties had an impact on rhizosphere and bulk soil communities. RDMC of plant roots is one indicator, reflecting the resource use strategy, i.e., resource acquisition and conservation (Prieto et al., 2015), and is therefore strongly linked to soil nutrient availability (Legay et al., 2014). Moreover, root chemistry and nutrient concentration have positive effects on carbon and nitrogen cycling (Bardgett et al., 2014) and probably control the nutrient abundances in soil (Carrillo et al., 2017). This, in turn, may lead to changes in microbial communities. Intriguingly, root nitrogen, which is often considered a good proxy for the nutrient status in soils (Bardgett et al., 2014), was also a significant driver of both microbial communities in the present study. Furthermore, root Mg for bacteria and Ca concentration for fungi were found to be important in shaping their communities. Root Ca and Mg are key for root growth and elemental uptake (Fageria, 2009), and the latter is also known to be an essential element for microbial growth (Huber and Jones, 2013). Hence, there is the potential that microbial communities are indirectly influenced by root-induced changes in the soil nutrient solution. The results are indicators of the value of root traits in predicting soil processes, but also reinforce their significance in shaping microbial communities to consider them in future studies.

### Influence of Land-Use Intensity and Other Plot Variables on Microbial Soil Communities

There were no significant responses in microbial alpha-diversity to increasing LUI. However, as hypothesized, LUI was a strong driver of bacterial and fungal community composition in both the rhizosphere and the bulk soil. Similar results were obtained in a study on arbuscular mycorrhizal fungi in roots of grassland plants in Hainich-Dün (Vályi et al., 2015). Yet, these findings challenge previous studies by Herold et al. (2014) who found no correlation between microbial community composition with LUI in grassland topsoil from the Biodiversity Exploratories using phospholipid fatty acid profiles (PLFA profiles) and Kaiser et al. (2016) who found similar results for bacteria in grassland soils in Hainich-Dün. The authors argued that LUI is composed of interacting land-use effects of fertilization and perturbation via mowing and grazing activities. This rather accounts for the quantity and not for the type of the fertilizers (Blüthgen et al., 2012). The intensively managed experimental grassland plots in the Hainich-Dün Exploratory predominantly receive mineral fertilizers. Studies in agro-ecosystems have also revealed clear differences between the composition of bacterial and fungal soil communities associated with mineral or organic long-term fertilization (Hartmann et al., 2014; Francioli et al., 2016). Thus, the contrast between our study and the ones of Herold et al. (2014) and Kaiser et al. (2016) may be explained by the fact that they undertook their sampling in spring, while ours took place in autumn when the sum effects of the differential fertilization and all disturbances caused by mowing and grazing have more impact than before the vegetation period. It is noteworthy that, fungi have a larger niche range regarding LUI because they are associated to different LUI as function of the soil compartment and the plant functional group. In contrast, the response of bacteria was more consistent and they were specific to a particular LUI; this is consistent with Lauber et al. (2013). For example, the bacterial genera *Pseudonocardia* and *Skermanella* had a clear preference to intensive and the genera *Lysobacter* and *Bacillus* to extensive land-use but showed hardly any variation within the soil compartments and plant functional groups. For *Pseudonocardia* and *Skermanella* it was shown in farming systems that these genera were associated to systems with a stronger anthropogenic impact (Li et al., 2012). Members of *Lysobacter* appeared abundant in soil suppressive to root pathogens (Ciancio et al., 2016) and are known as chitinolytic bacteria (Lupatini et al., 2017), while the genus *Bacillus* has plant growth-promoting abilities (Ciancio et al., 2016). We found bacteria involved in chitinolysis and nitrogen fixation predominantly at low LUI (Supplementary Figure S9). Different observations were made for the fungal genera *Chaetomium* (molds) and *Trichosporon* (yeasts) with a preference to high LUI. *Chaetomium* seem to tolerate a high LUI more in the rhizosphere of grasses while *Trichosporon* in both soil compartments of grasses. *Chaetomium* species are considered as biocontrol agents in plant disease by the production of antimicrobial substances (Liu and Chang, 2018) and their abundance might be connected to the abundance of (plant) pathogens at a higher LUI here (Supplementary Figure S9). Furthermore, fungal genera with a preference to low LUI like *Cuphophyllus* and *Camarophyllopsis* also have a high variability within the treatments. In particular, an effect of the plant functional groups was indicated for *Cuphophyllus* and *Camarophyllopsis*. Both genera are known for their preference for nutrient limited meadows and their sensitivity to nitrogen inputs (Öster, 2008; Lodge et al., 2014) and seem to vary this preference through the plant functional groups. This differential variability may mirror differences in the life strategies and growth forms in relation to niche differentiation in bacteria versus fungi (Boer et al., 2005). Most bacteria are present as individual cells and fast-growing with low C use efficiency whereas fungi exhibit a hyphal growth form and have rather a slow growth (van der Heijden et al., 2008; Strickland and Rousk, 2010). Bacteria may outcompete the slow growing filamentous fungi especially under high resource availability and LUI which lead to shifts of the fungal niche. Fungi, in turn, are able to translocate resources from microsites where they are present to sites where they are restricted (Strickland and Rousk, 2010) and thus, they can react more flexible to changes in resources availability and to competition. Furthermore, bacteria are expected to have higher nutrient requirements than fungi which leads to the dominance of bacteria under high LUI (N availability) and presence of easily degradable organic compounds (root exudates, Güsewell and Gessner, 2009). Yet, for most of the bacterial and fungal genera discovered here, we have a limited knowledge about their ecological roles. However, for those few genera with known ecological roles, information mostly derive from a subset of well-studied taxa within a given genus and thus, may not necessarily apply to all phylogenetic related members (Louca et al., 2016a; Nguyen et al., 2016). Consequently, it would be more accurate to assign ecological roles only from knowledge about microbial species to avoid bias due to intergeneric variation (Nguyen et al., 2016). It would be of great interest to gain additional information, e.g., by analyzing the distribution of genes that are relevant for important ecophysiological functions (Hartmann et al., 2014) and to relate them to the microbial genera or species distribution patterns along with LUI.

Furthermore, the soil edaphic properties such as soil C/N ratio, plant available- and soil total phosphorus were strongly correlated with LUI and together with soil type (Berg and Smalla, 2009) they are widely reported to control the distribution of microbial communities (Lauber et al., 2008; Thomson et al., 2015; Fierer, 2017). Consistent with previous reports (Lauber et al., 2009; Kaiser et al., 2015), we found soil pH to be one of the strongest factors shaping bacterial communities and to a lesser extent those of fungi. Moreover, both microbial groups were strongly influenced by the C/N ratio in the rhizosphere as well as in the bulk soil. This has been also demonstrated by others (Kuramae et al., 2012; Hermans et al., 2017) and suggests the importance of soil nutrient pools. In particular, we found phosphorus to play a substantial role; indeed, the fungal community is strongly driven by plant available phosphorus (Lauber et al., 2008; Tian et al., 2017). Together with the markedly lower explained proportion of the plot variables for fungi compared to bacteria, this again supports the hypothesis that the two microbial groups react differently to environmental conditions.

## Conclusion

Our study provided evidence for a general rhizosphere effect on communities of bacteria and fungi with enhanced diversity of functional groups such as copiotrophs or plant growth-promoting taxa. Although it is often reported in the literature that the rhizosphere is less diverse than the bulk soil, we found higher microbial diversity in the rhizosphere. Because we sampled at the end of the vegetation season, further studies at different stages are required. Plant functional group and species identity therein did not significantly affect the community composition of bacteria and fungi, suggesting either a high equivalence in exudates or the requirement for longer effects than in our 6 months phytometer study. In contrast, root traits were strong drivers of microbial composition. In addition, LUI coupled with plant productivity, neighboring plant richness and soil chemical properties, especially soil C/N ratio, had a major impact on the microbial community composition in both soil compartments and appeared to explain a large part of the variation. While fungal taxa were highly flexible to varying LUI, bacterial genera were more specific. Overall, our study indicates that functional groups of plants are weak indicators of the microbial communities encountered in their rhizosphere and bulk soil, while root traits, land-use and soil conditions matter much more. This suggests the need to place more emphasis on the root traits and land-use intensity to unravel the link between below and aboveground communities and their drivers.

## Data Availability

All generated and analyzed datasets for this study can be found in the BExIS platform (https://www.bexis.uni-jena.de/).

## Author Contributions

FB, HB, and TW conceived and designed the experiments. KH and RS performed the field experiments. RS performed the laboratory works. IS contributed data. RS, KG, and KH wrote the manuscript, with input from IS, GL, TW, HB, and FB. RS, KG, and GL analyzed and interpreted the results. FB, HB, and TW obtained funding. All authors contributed to revisions and gave approval for submission.

## Conflict of Interest Statement

The authors declare that the research was conducted in the absence of any commercial or financial relationships that could be construed as a potential conflict of interest. The reviewer DF declared a past co-authorship with several of the authors FB, GL, and TW to the handling Editor.
