# Supplementary material for: Land-Use Intensity Rather Than Plant Functional Identity Shapes Bacterial and Fungal Rhizosphere Communities
Source: Front Microbiol. 2018 Nov 20;9:2711. doi: 10.3389/fmicb.2018.02711 (PMC6255942; doi:10.3389/fmicb.2018.02711)
Supplement: Supplementary file 1 [file Data_Sheet_1.pdf]

Supplementary Material

Land-use intensity rather than plant functional identity shapes bacterial and fungal rhizosphere communities

Ricardo Schöps[*], Kezia Goldmann, Katharina Herz, Guillaume Lentendu, Ingo Schöning, Helge Bruelheide, Tesfaye Wubet, François Buscot

*** Correspondence:** Ricardo Schöps: ricardo.schoeps@ufz.de

1 Supplementary Data

1.1 Supplementary Figures

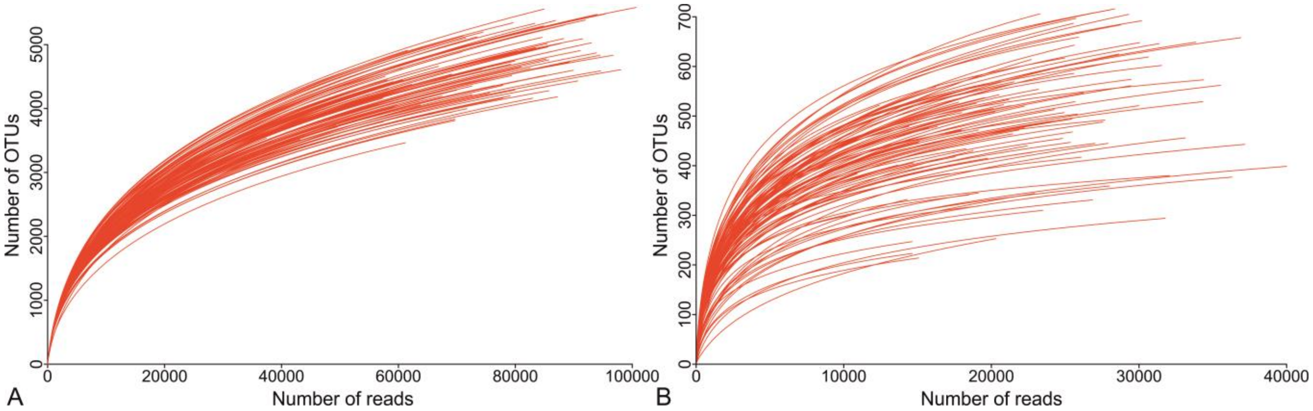

Supplementary Figure S1. Individual rarefaction curves of bacterial (A) and fungal (B) OTUs at a 97 % similarity level of all 104 soil samples.

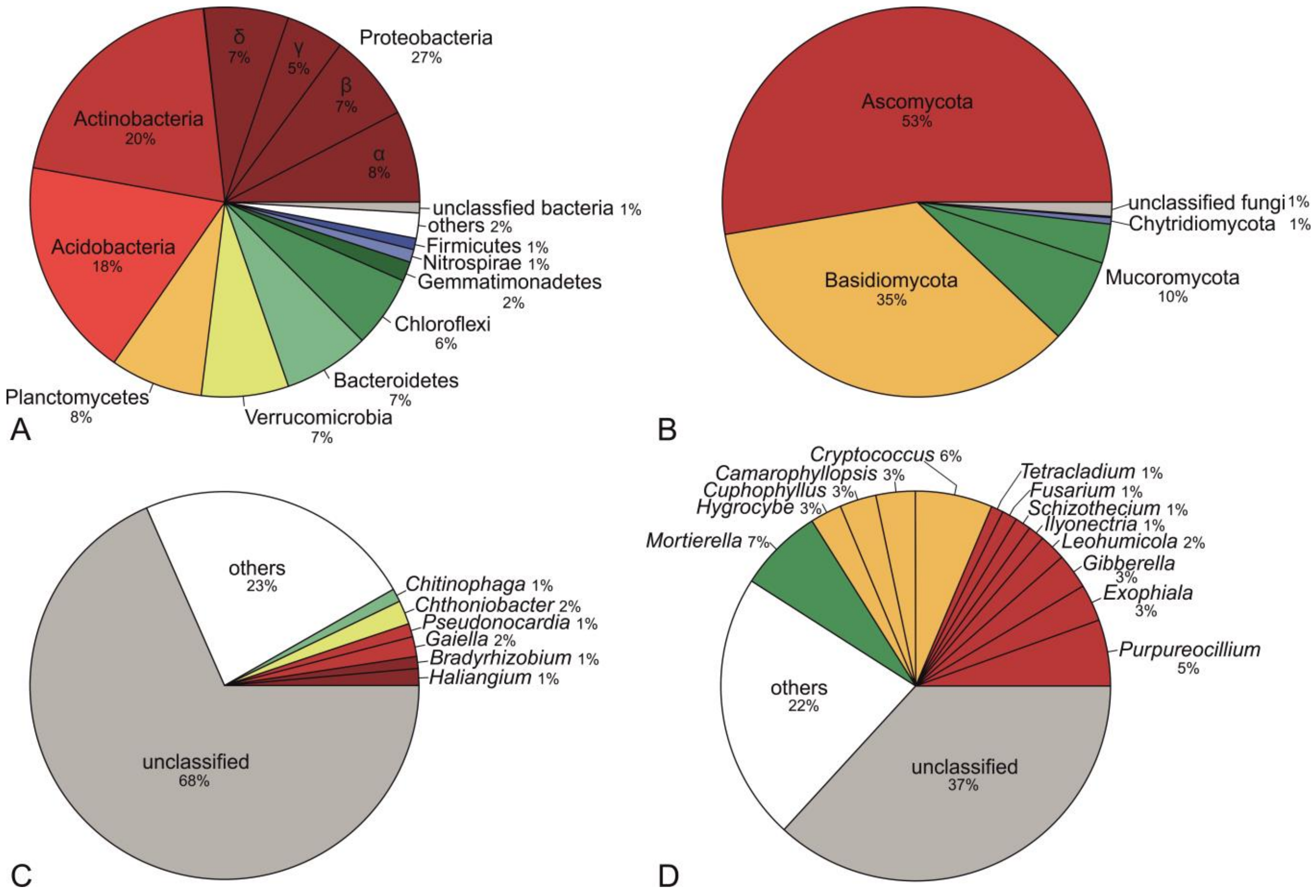

Supplementary Figure S2. Bacterial (A and C) and fungal (B and D) OTU relative abundances of the most abundant phyla and genera (i.e. with at least 1 % relative abundance). Phyla and genera with a relative OTU abundance less than 1% were grouped into “others”. (A) the proteobacterial phylum is broken down into classes. (B) Ascomycota fungi could be further divided into the growth morphological types ascomycetes mold (8 %), yeast fungi (4 %) and other ascomycetes (41 %) and Basidiomycota fungi into basidiomycetes yeast (7 %), agaricoid (7 %) and other basidiomycetes (21 %). Mucoromycota fungi (green) are mainly comprised of the subphyla Mortierellomycotina (7 %) and Glomeromycotina (3 %). (C and D) the color of an abundant bacterial or fungal genus corresponds to the respective color of the bacterial or fungal phylum.

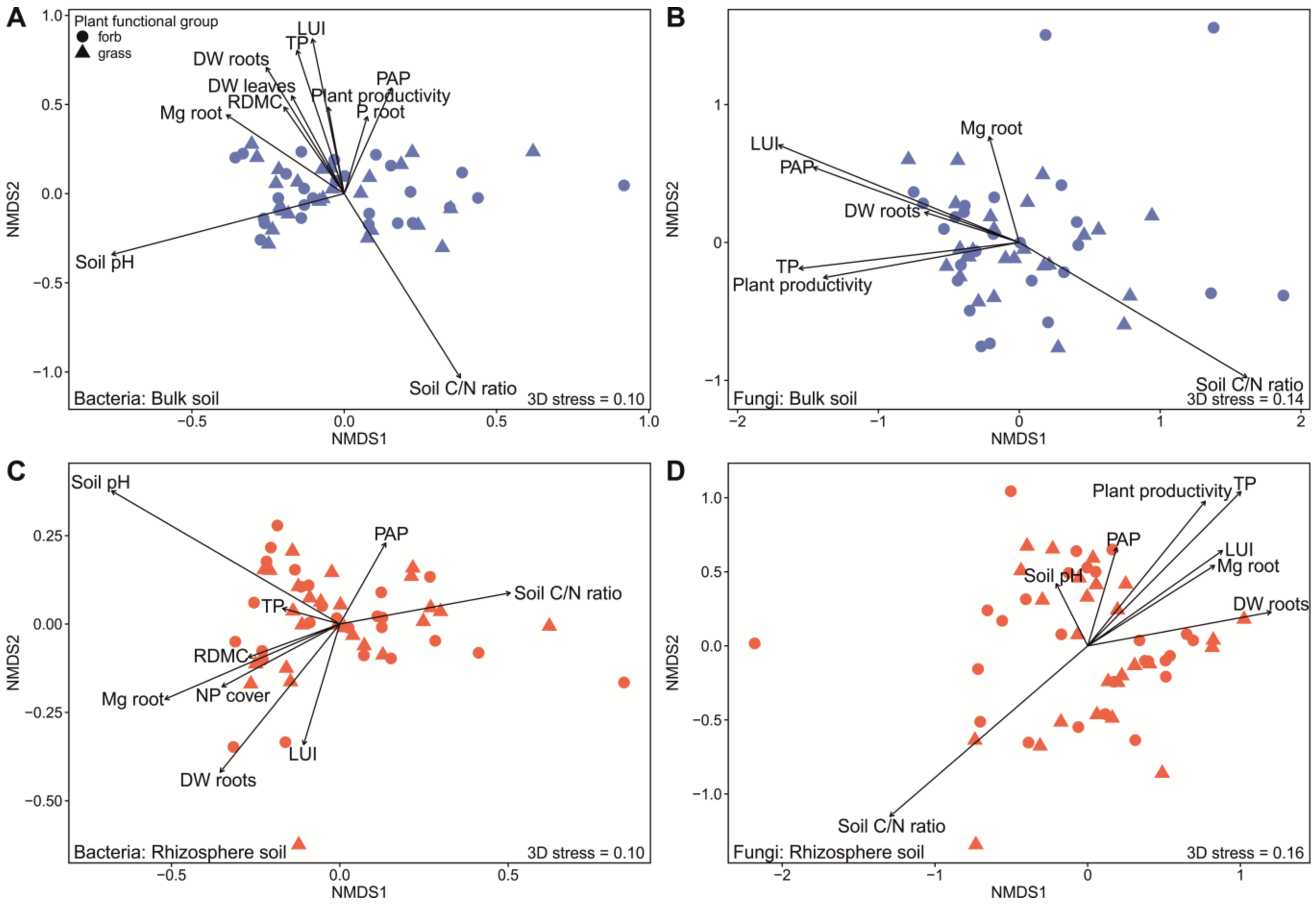

Supplementary Figure S3. Nonmetric multidimensional scaling (NMDS) ordination of bacterial (A and C) and fungal (B and D) community composition in bulk (blue) and rhizosphere (red) soil both under forbs (●) and grasses (▲) based on Bray-Curtis dissimilarity and k = 3 dimensions. Significant vectors ($p < 0.05$) correlated with community composition are shown. LUI, land-use intensity index; DW leaf/root, dry weight root/leaf; RDMC, root dry matter content; Mg root, root magnesium content; P root, root phosphorus content; Soil C/N ratio, soil carbon to nitrogen ratio; PAP, plant available phosphorus; TP, soil total phosphorus; Plant productivity, plant biomass per plot; NP cover, /cover of the neighboring plants.

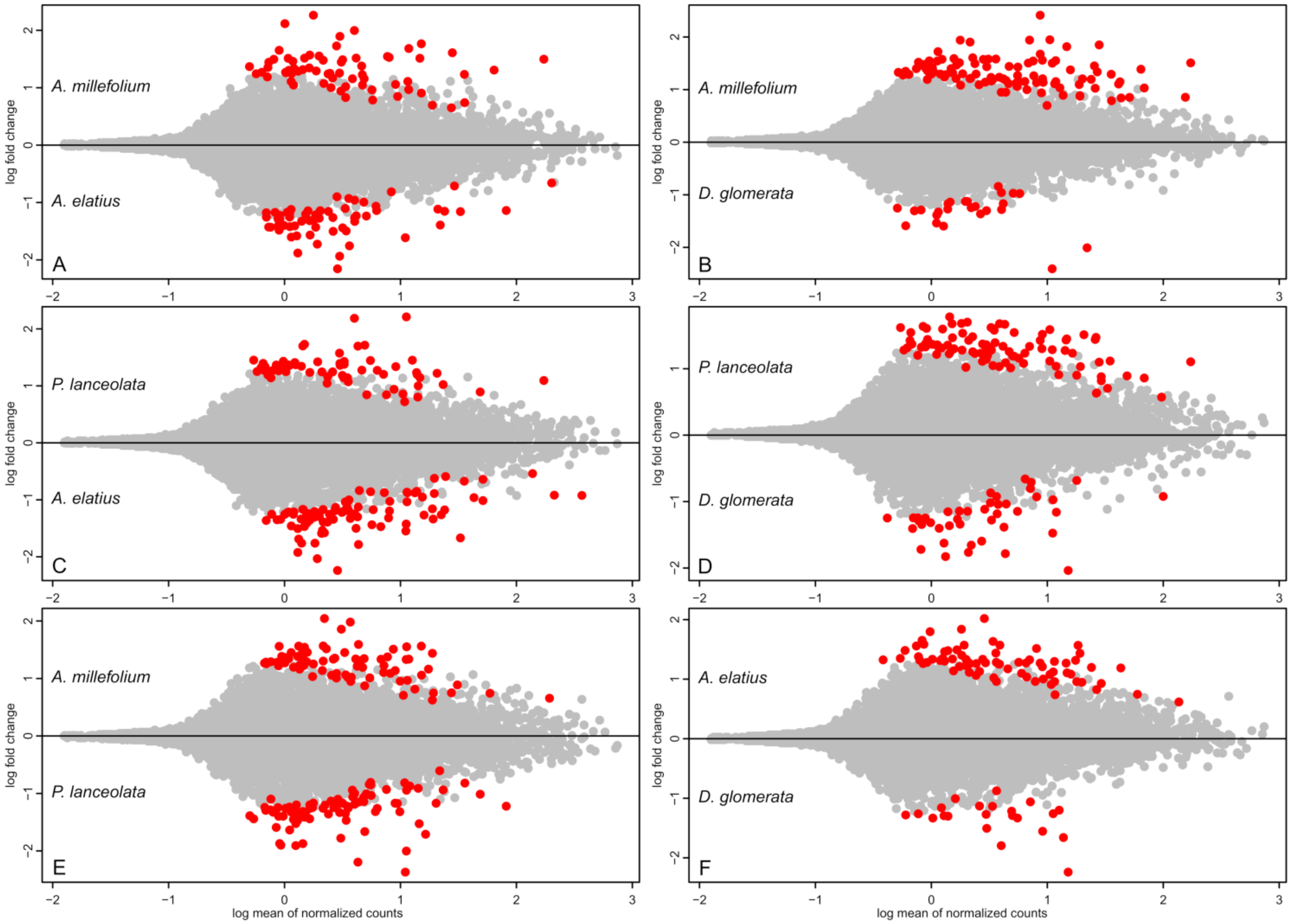

Supplementary Figure S4. Pairwise comparisons of the plant phytometer species in rhizosphere soil for bacterial OTUs. In each experimental plot, the shapes represent individual OTUs whose position on the x-axis reflects their abundance (normalized counts) and position on the y-axis represents the fold change in the indicated comparison. All bacterial OTUs significantly differentially represented between two plant species are highlighted (Wald test, $p < 0.05$; all bacterial significantly different OTUs ($p < 0.05$) are displayed with taxonomic affiliation in Supplementary Table S7).

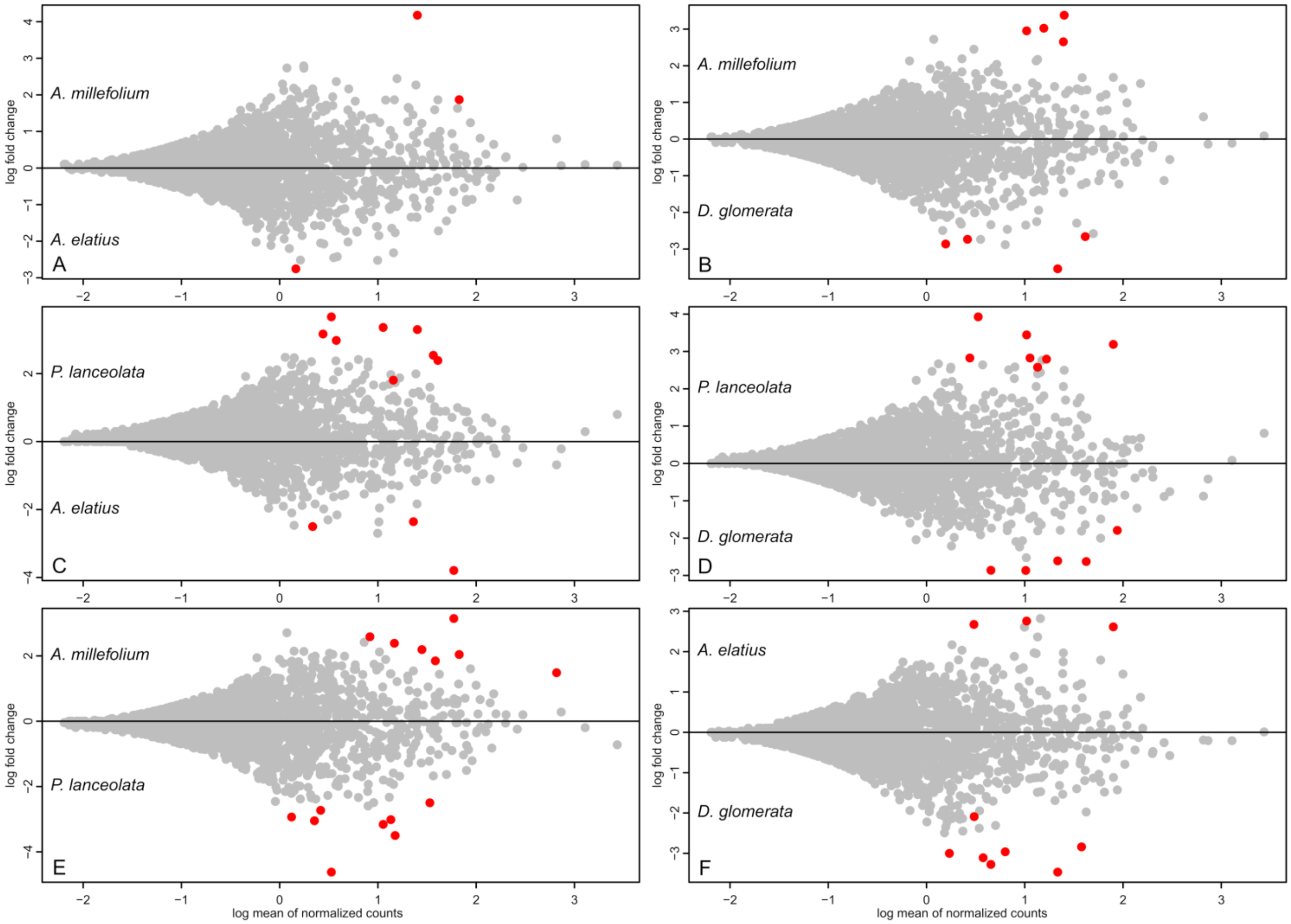

Supplementary Figure S5. Pairwise comparisons of the plant phytometer species in rhizosphere soil for fungal OTUs. In each experimental plot, the shapes represent individual OTUs whose position on the x-axis reflects their abundance (normalized counts) and position on the y-axis represents the fold change in the indicated comparison. All fungal OTUs significantly differentially represented between two plant species are highlighted (Wald test, $p < 0.05$): all fungal significantly different OTUs ($p < 0.05$) are displayed with taxonomic affiliation in Supplementary Table S7).

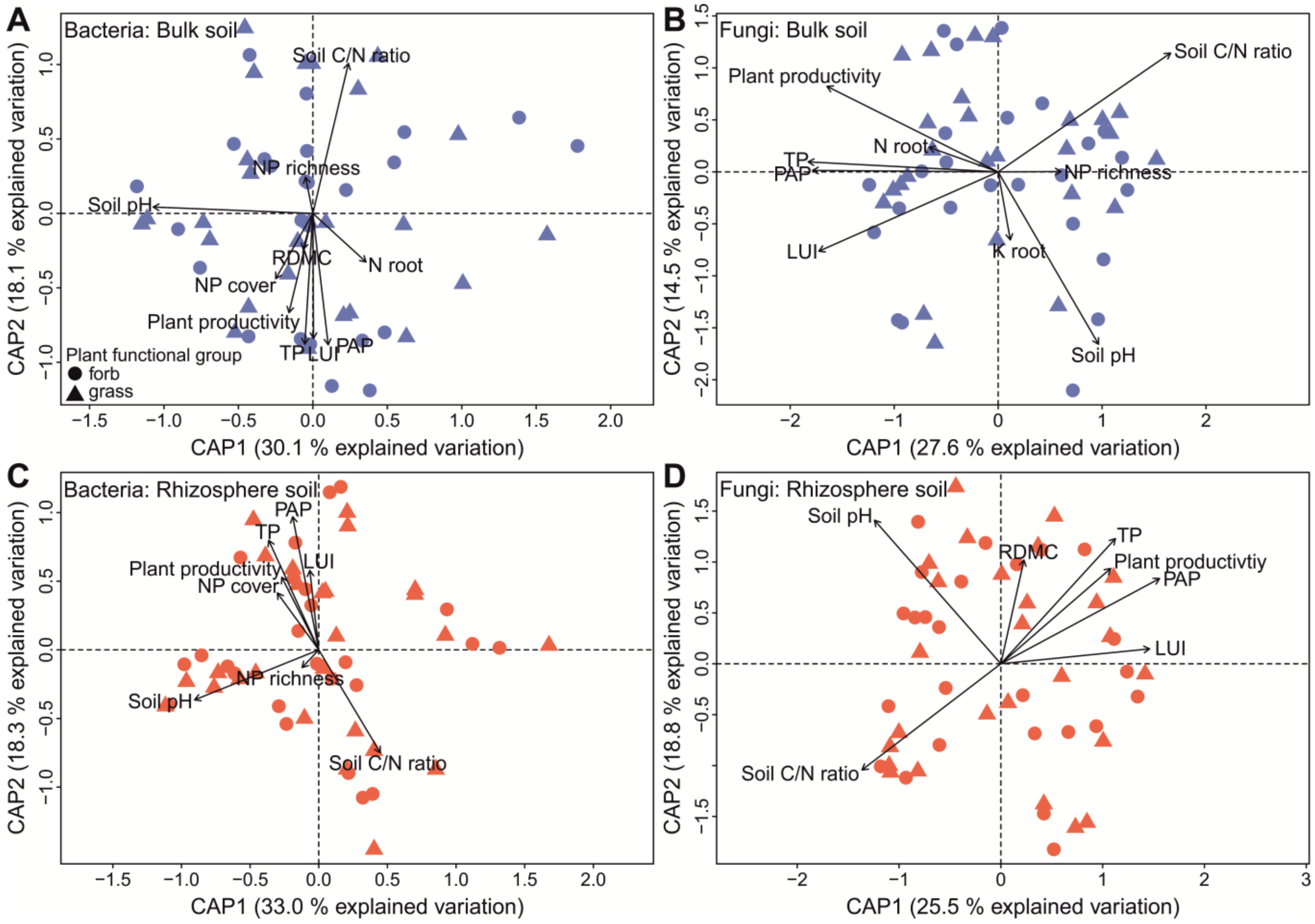

Supplementary Figure S6. Partial distance-based redundancy analysis (db-RDA) for the bacterial (A and C) and fungal (B and D) communities in the bulk and rhizosphere soil associated with the plant traits and environmental factors based on Bray-Curtis dissimilarity. LUI, land-use intensity index; RDMC, root dry matter content; N root, root nitrogen content; K root, root potassium content; Soil C/N ratio, soil carbon to nitrogen ratio; PAP, plant available phosphorus; TP, soil total phosphorus; Plant productivity, plant biomass per experimental plot; NP richness/cover, richness/cover of the neighboring plants.

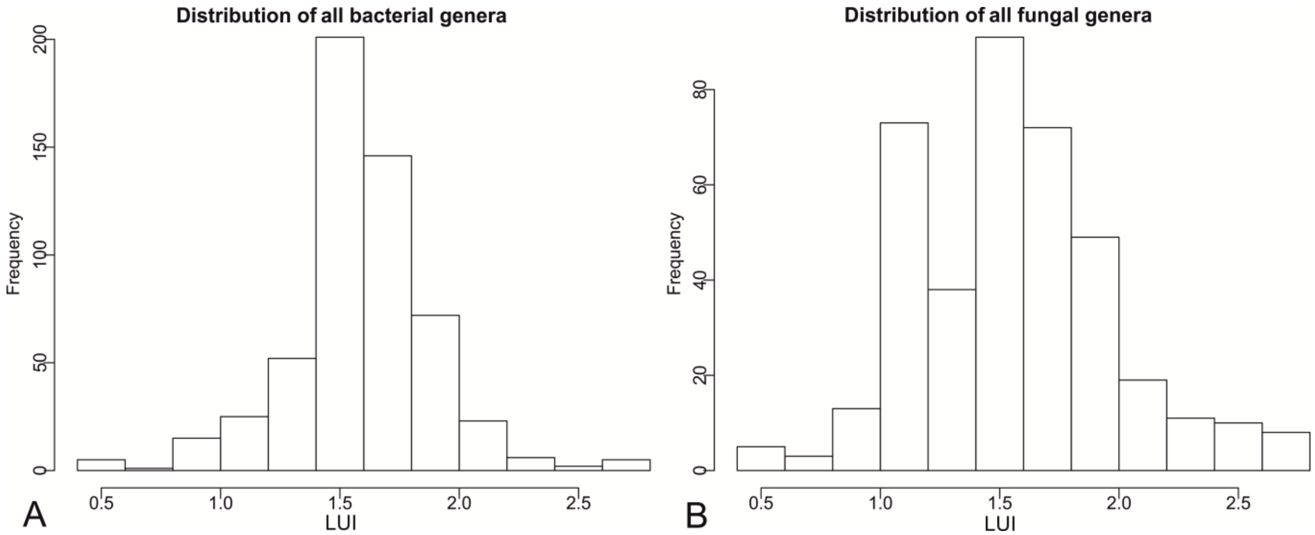

Supplementary Figure S7. Histogram showing frequency in abundance-weighted mean LUI of all 553 bacterial (A) and 393 fungal (B) genera.

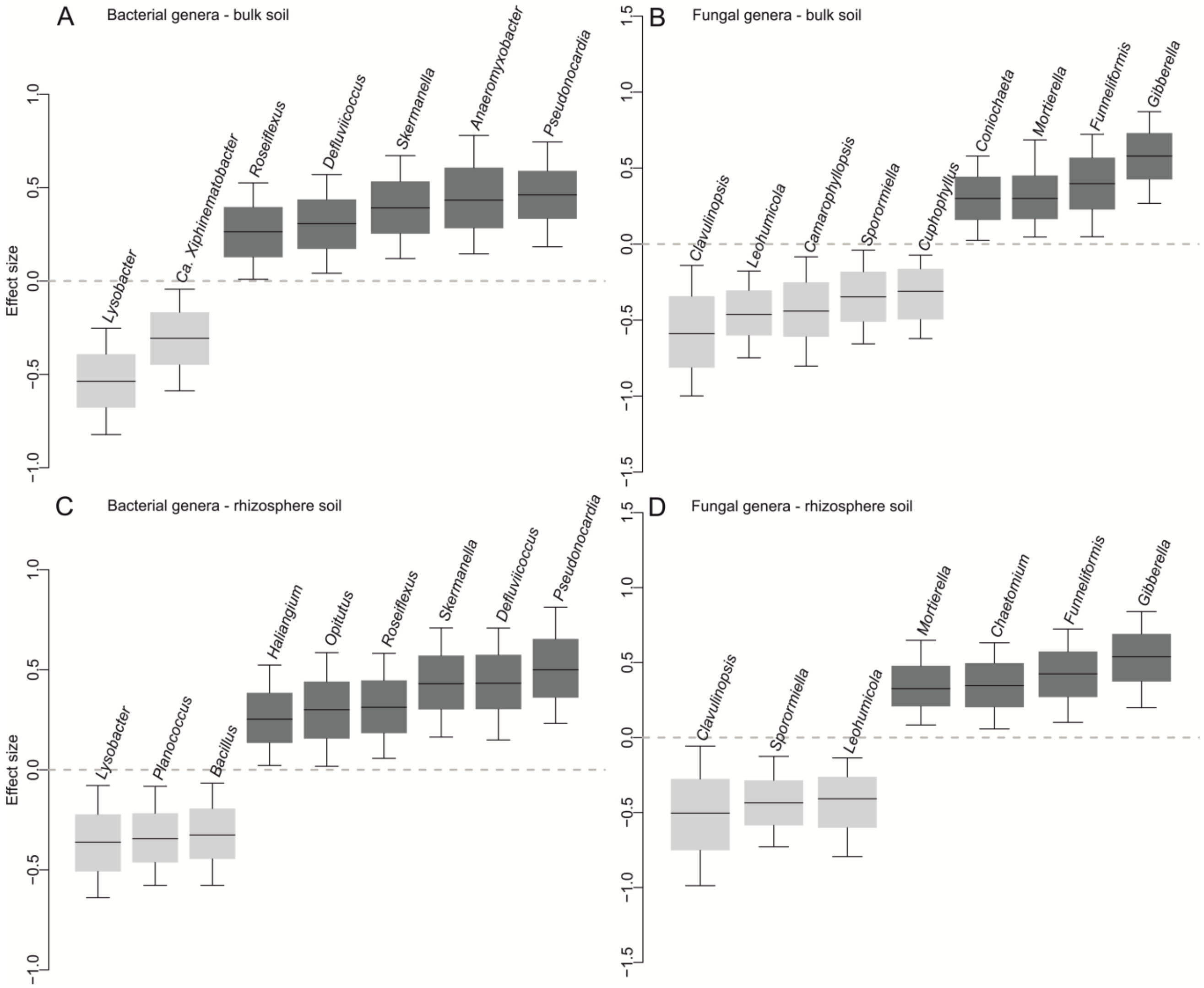

Supplementary Figure S8. Coefficient plots of the generalized joint attribute modeling (gjam) analysis. Effect of land-use intensity (LUI) index on the composition count data of the top ranked bacterial (A-C) and fungal (B-D) genera from the SIMPER analysis with the strongest response to LUI. Posterior distributions for microbial genera responses to LUI are well identified, with narrow 95 % credible intervals; genera indicated in dark grey exhibited a strong positive and light grey a strong negative response to LUI.

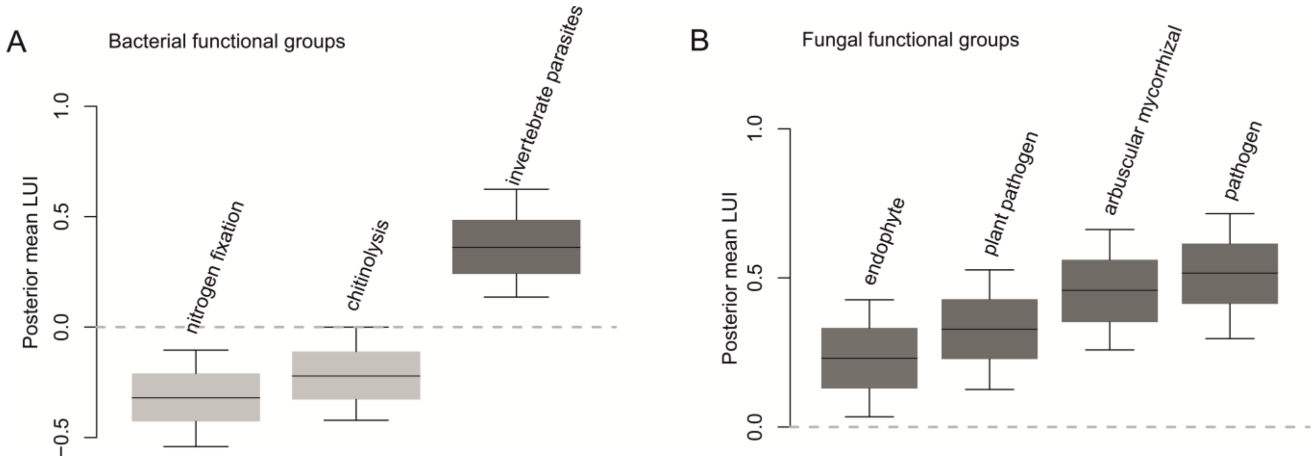

Supplementary Figure S9. Coefficient plots of the generalized joint attribute modeling (gjam) analysis. Relative abundances of bacterial (A) and fungal (B) functional groups in response to land-use intensity (LUI) index were tested and functional groups with a strong response are shown. Posterior distributions for microbial functional groups responses to LUI are well identified, with narrow 95 % credible intervals; functional groups indicated in dark grey exhibited a strong positive and light grey a strong negative response to LUI.

1.2 Supplementary Tables

Supplementary Table S1. Values of environmental variables in each experimental plot. LUI, land-use intensity index of 2014; Soil pH, pH of soil; Soil C/N ratio, soil carbon to nitrogen ratio; PAP, plant-available NaHCO3-extractable phosphorus; TP, total soil phosphorus concentration; Plant productivity, plant biomass per experimental plot.

Plot	*LUI*	*Soil pH*	*Soil C/N ratio*	*PAP*	*TP*	*Plant productivity*	*Management*	*Treatment*	*Soil type*
HEG01	2.530	6.520	9.882	22.987	1.030	511.100	mown pasture	fertilized	Cambisol
HEG03	2.660	7.100	8.910	16.789	1.230	340.650	mown pasture	fertilized	Vertisol
HEG05	2.150	6.920	9.586	14.761	0.770	230.350	mown pasture	fertilized	Stagnosol
HEG09	0.930	6.770	11.109	9.938	0.580	241.850	pasture	non-fertilized	Stagnosol
HEG14	1.840	6.360	10.745	18.686	1.250	431.450	mown pasture	fertilized	Stagnosol
HEG16	1.060	6.830	10.098	12.944	1.150	331.550	pasture	non-fertilized	Stagnosol
HEG21	0.580	7.090	10.043	30.048	0.910	218.200	pasture	non-fertilized	Stagnosol
HEG26	1.750	7.170	10.058	12.386	0.820	229.750	meadow	fertilized	Cambisol
HEG27	1.710	7.140	10.107	13.023	1.110	481.400	meadow	fertilized	Cambisol
HEG33	1.560	5.010	10.499	47.416	0.490	234.100	mown pasture	fertilized	Cambisol
HEG40	1.590	6.550	9.642	39.949	1.320	280.750	pasture	non-fertilized	Cambisol
HEG41	1.100	7.060	11.174	7.829	0.710	249.600	pasture	non-fertilized	Cambisol
HEG42	1.060	7.100	10.723	13.874	0.660	109.800	pasture	non-fertilized	Cambisol

Supplementary Table S2. Information about the plant traits, plot variables and local neighborhood vegetation used. The last column shows the plant traits and plot variables used for the variation partitioning. NP richness/cover, richness/cover of the neighboring plants.

Abbreviation	*Unit*	*Description*	*Predictor type*
DW leaves	mg	Dry mass	Plant trait
DW shoots	g	Dry mass	Plant trait
DW roots	g	Dry mass	Plant trait
RDMC	mg/g	Root dry mass per root fresh mass	Plant trait
Rvol	cm^3	Root volume	Plant trait
Root C	%	Root carbon concentration	Plant trait
Root N	%	Root nitrogen concentration	Plant trait
Root P	µmol/g	Root phosphorus concentration	Plant trait
Root Mg	µmol/g	Root magnesium concentration	Plant trait
Root Ca	µmol/g	Root calcium concentration	Plant trait
Root K	µmol/g	Root potassium concentration	Plant trait
LUI		Land-use intensity index	Plot variable
Soil pH		pH of soil	Plot variable
Soil C/N ratio		Soil carbon to nitrogen ratio	Plot variable
PAP	mg/kg	Plant available phosphorus concentration	Plot variable
TP	g/kg	Total soil phosphorus concentration	Plot variable
Plant productivity	g/m^2	Weight of dried biomass	Plot variable
NP richness		Plant species richness measured around each phytometer	
NP cover	%	Grass and herb cover around each phytometer	

Supplementary Table S3. An XLSX table contains bacterial and fungal genera that contribute to the observed difference in the bacterial and fungal communities of the bulk and rhizosphere soil assemblages based on similarity percentage (SIMPER) analysis.

Supplementary Table S4. An XLSX table contains Spearman's rank correlation matrix and correlation significance of relevant variables.

Supplementary Table S5. Diversity indices for bacterial and fungal communities in bulk (BS) and rhizosphere soil (RS). Significant differences between soil compartments are indicated by lowercase letters according to Tukey HSD post hoc test ($p < 0.05$). Abundance-based coverage estimator (ACE).

Diversity indices	*Bacteria*		*Fungi*	
	BS	*RS*	*BS*	*RS*
Richness	2925.08 (±228.93)^{b}	3073.86 (±214.94)^{a}	335.21 (±81.62)^{b}	407.85 (±63.70)^{a}
Shannon diversity	6.79 (±0.12)^{b}	6.86 (±0.15)^{a}	3.70 (±0.90)^{a}	3.91 (±0.65)^{a}
Evenness	0.85 (±0.01)^{a}	0.86 (±0.01)^{a}	0.63 (±0.14)^{a}	0.65 (±0.10)^{a}
ACE	3310.29 (±279.78)^{b}	3491.13 (±262.54)^{a}	361.81 (±87.54)^{b}	446.72 (±70.73)^{a}

Supplementary Table S6. Results of the LMEM for OTU richness and ACE of bacteria and fungi. For the response variables bacterial and fungal OTU richness and abundance-based coverage estimator (ACE), the model with the lowest AICc is shown (where no significant interactions were found). The full model contained soil compartment, land-use intensity (LUI) and plant functional group (PFG)/plant species (PS) as fixed factors, as well as all possible interactions. Experimental plot and soil type were random factors. There were 104 observations. Marginal R^2 indicates how much variance is explained in the model without random factors, conditional R^2 is how much variance is explained by the model including random factors. ***p < 0.001; n.s., not significant.

LMEM – PFG	*Bacteria*				*Fungi*			
	Richness		*ACE*		*Richness*		*ACE*	
	Estimate	*p*	*Estimate*	*p*	*Estimate*	*p*	*Estimate*	*p*
Intercept	2853.79	***	3238.73	***	322.984	***	349.563	***
Soil compartment	148.79	***	180.84	***	72.635	***	84.905	***
LUI	36.06	n.s.	32.36	n.s.	3.318	n.s.	3.108	n.s.
Grasses	28.71	n.s.	40.96	n.s.	13.981	n.s.	14.685	n.s.
R^2 marginal	0.12		0.11		0.21		0.23	
R^2 conditional	0.39		0.43		0.50		0.53	
Pairwise comparison	Mean ± SD		Mean ± SD		Mean ± SD		Mean ± SD	
Bulk soil	2925 ± 228[b]		3310 ± 280[b]		337 ± 82[b]		362 ± 88[b]	
Rhizosphere soil	3073 ± 215[a]		3491 ± 263[a]		410 ± 64[a]		447 ± 71[a]	
LMEM – PS	*Bacteria*				*Fungi*			
	Richness		*ACE*		*Richness*		*ACE*	
	Estimate	*p*	*Estimate*	*p*	*Estimate*	*p*	*Estimate*	*p*
Intercept	2843.41	***	3220.12	***	328.369	***	355.09	***
Soil compartment	148.79	***	180.84	***	72.635	***	84.905	***
LUI	36.06	n.s.	32.36	n.s.	3.318	n.s.	3.108	n.s.
Species_Plan.lanc	20.77	n.s.	37.22	n.s.	−10.769	n.s.	−11.054	n.s.
Species_Arrh.elat	19.08	n.s.	33.74	n.s.	11.346	n.s.	13.018	n.s.
Species_Dact.glom	59.12	n.s.	85.39	n.s.	5.846	n.s.	5.297	n.s.
R^2 marginal	0.12		0.12		0.21		0.24	
R^2 conditional	0.40		0.43		0.51		0.54	

Supplementary Table S7. An XLSX table contains two tables of all bacterial and fungal OTUs with significant fold change ($p < 0.05$) between both plant functional groups (PFG) and the four plant species (PS). These tables correspond to Figure 6 as well as Supplementary Figure S3 and S4.
